# An evaluation of strategies commonly used by health advocate programs

**DOI:** 10.1371/journal.pone.0350645

**Published:** 2026-07-17

**Authors:** Jingyao Huang, Diwakar Gupta

**Affiliations:** 1 Bloch School of Business, University of Missouri-Kansas City, Kansas City, Missouri, United States of America; 2 McCombs School of Business, University of Texas, Austin, Texas, United States of America; University of Porto Faculty of Medicine: Universidade do Porto Faculdade de Medicina, PORTUGAL

## Abstract

Many non-urgent and routine medical procedures, such as MRI and CT Scans, are performed under standardized industry protocols with minimal process variations. However, large price variations exist among providers offering these services within the same geographic region. Many insurers and independent vendors have implemented health advocate/concierge programs to steer beneficiaries to high-quality and low-cost providers. These programs also aim to reduce costs. The Blue Cross Blue Shield (BCBS) of Texas’ Benefits-Value-Advisor (BVA) program is one of the most cited examples. It uses three key strategies: recommendation, unconditional monetary rewards, and persuasion. However, their effectiveness in influencing beneficiaries’ choices, particularly for routine medical procedures, has not been tested. This study fills the gap through a behavioral experiment, which provides insights into how beneficiaries may respond to these strategies and providing guidance for health plan managers. A full-factorial-between-subjects experiment was conducted with 500 subjects recruited through Amazon Mechanical Turk. The experiment design included three treatments: recommendation, copay waiver, and persuasion, each with two levels (Yes or No), resulting in eight distinct scenarios. Subjects were randomly assigned to one of eight scenarios. The survey data were analyzed using a logit regression model. We found that subjects who received recommendations were 32.7% more likely to select the lowest-cost provider and had a 27.3% greater chance of choosing lower-cost providers. However, the effectiveness of recommendations diminished when subjects mistrusted their insurance companies. Neither copay waiver nor persuasion had a significant impact on provider choice. We acknowledge that these findings are derived from a hypothetical experimental setting using an online sample, and that actual beneficiary behavior in clinical settings may differ due to additional factors such as physician referrals, appointment availability, urgency of medical needs and convenience. Our results should be interpreted as providing suggestive evidence rather than directly generalizable predictions of real-world behavior.

## Introduction

Many non-urgent and routine medical procedures, such as MRI and CT Scans, adhere to standardized industry protocols, resulting in minimal variation in quality across providers. However, large price variations exist among providers that offer these services within the same geographic region [1,2]. For example, in Austin, Texas area, the price of a cervical spine MRI procedure, varied from $869 to $5,955 in 2025 [3]. These characteristics of the health services market lead to high costs for insurers, while beneficiaries do not necessarily receive higher quality care. Insurers in recent years have implemented strategies to encourage beneficiaries to utilize high-value (low-cost, high-quality) care providers. Similar programs are also offered by independent vendors. This study concerns a health advocate program called the Benefits-Value-Advisor (BVA) program, which has been introduced by the Blue Cross Blue Shield (BCBS) of Texas on behalf of self-insured employers like the University of Texas (UT). It is one of the most cited insurer-led program of its type. The program targets non-urgent, shoppable medical services such as MRI and CT scans, colorectal screenings, and ultrasounds, with the goal of lowering long-term health plan costs for such procedures [4]. We provide a summary table of other similar programs by insurers and independent vendors in S1 File. The BVA program steers beneficiaries to utilize high-value providers, thereby putting pressure on all the providers to reduce prices and improve quality.

While the BVA program has utilized recommendation, copay waiver, and persuasion to achieve its goal, the effectiveness of strategies it employs remains untested. The authors obtained aggregate data from BCBS for MRI and CT scans, which is described in S2 File. The data show that beneficiaries choose lower cost providers after the program was implemented. However, the data are not sufficiently granular to allow a comparison of the effectiveness of different strategies, either independently or in combination with other strategies. This paper concerns an online experiment that was conducted through Amazon’s Mechanical Turk (MTurk) platform to evaluate the effectiveness of the three approaches commonly used by BVA agents to affect beneficiaries’ choices.

The goal of this study is to provide insights into how insurer-led interventions may influence beneficiary decision-making in BVA-like health advocate programs, thereby filling a gap in knowledge. The specific research questions that this study answers are as follows.

- Keeping provider quality and beneficiaries’ out-of-pocket costs the same across all providers, what proportion of beneficiaries change their initial choice of provider by switching to either the lowest-cost or one of lower-cost providers?
- Which of the three strategies, either independently or in combination with others, is most effective in increasing the proportion of beneficiaries switching to lower-cost providers?
- Among individual characteristics, such as beneficiaries’ familiarity with health insurers costs, and their level of trust of their insurers, which factors have the greatest impact on beneficiaries’ choices?
- What insights do the experiment’s results provide for improving the effectiveness of BVA-like programs?

**Fig 1:**
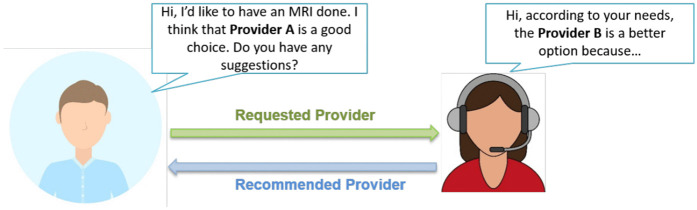
An example of the interaction between an agent and a beneficiary in the BVA program.

The functioning of the BVA program is illustrated in Fig 1. Beneficiaries often select high-cost providers, in part because such providers have high brand visibility, while BVA agents steer them toward low-cost options (see S2 File for details). Beneficiaries are encouraged to utilize the BVA program by calling a program agent (hereafter *agent*) before selecting their service providers for non-urgent medical procedures. They may have an initial preference for a provider, say Provider A, which is referred to as the *requested provider*. Based on the beneficiary’s needs, location, and travel constraints, the agent may recommend a different provider, say Provider B, referred to as the *recommended provider*. This type of intervention is referred to as the recommendation treatment in this paper. However, beneficiaries retain full autonomy: they may select the requested provider, the recommended provider, or an entirely different option, with no penalty for disregarding advice from the agent. The final choice is referred to as the *selected provider*.

BCBS has offered the BVA service to beneficiaries enrolled in the UT Select Plan since 2014. UT pays BCBS a fixed fee of $10 per member per month to subscribe to the BVA services, regardless of usage, and it is also responsible for the actual medical expenditures of the beneficiaries. As a self-insured employer, UT assumes the financial risk, while BCBS serves as the plan administrator. In this paper, the term *insurer* is used to refer to both entities. In addition to making recommendations, the BVA may offer copay waiver and/or utilize persuasion by testimonials. These strategies are described next.

UT Select beneficiaries typically pay a fixed copay (e.g., $100) for diagnostic imaging. Alternative payment structures are possible. Those are described in S3 File. To incentivize participation, BCBS waives the copay for plan members who consult an agent before selecting an MRI provider. This waiver is unconditional, i.e., beneficiaries receive it simply by using the BVA program, regardless of their chosen provider. It is offered only for MRI procedures. In addition to inducing a higher participation rate, insurers may anticipate that beneficiaries will reciprocate by selecting low-cost providers, offsetting the program’s expense. This type of intervention is referred to as the copay waiver treatment in this paper. Persuasion by testimonials is the third type of treatment. It entails the agents informing beneficiaries about the positive experience of other beneficiaries with the recommended providers. All three strategies are described in detail in the Methods section.

There have been attempts in the literature to explain the large spread in prices for shoppable medical services. It has been argued that either due to lack of awareness, or the absence of price transparency tools, or high search costs, beneficiaries are often unaware of the fees providers charge their insurers. Additionally, the moral hazard inherent in health insurance provides little incentive for beneficiaries to choose low-cost providers. Therefore, they may select providers based on the strength of their doctors’ referrals and brand recognition. Absent pressure from beneficiaries, prices are mostly influenced by factors such as market concentration and providers’ bargaining power in negotiations with insurers [5,6]. Viewed from this perspective, the BVA program helps bring about awareness of prices that insurers pay, reduces search costs, and employs a combination of recommendation, copay waiver, and persuasion to influence those who might not have a strong preference for any particular provider. There are also studies in the literature of other approaches used by insurers to lower costs for shoppable medical services. We describe those studies and their findings in comparison with the findings of our paper in the Discussion section. To the best of our knowledge, no previous study compares and contrasts the effectiveness of strategies utilized by the BVA program.

For standardized medical procedures, higher prices do not necessarily reflect higher quality. Prices are negotiated across a multitude of services that a provider offers. A provider may offer a low overall price for a bundle, e.g., bundled charges for knee joint replacement surgery, while having a high price for a particular procedure such as MRI scan that is included in the bundle. When an agent recommends a low-cost provider for a shoppable medical procedure, that provider is not necessarily an inferior option. It is likely that the provider has a lower negotiated price for that procedure (see S4 File for more details).

## Methods

### Ethical approval

This study was reviewed and exempted by the University of Texas (UT) Institutional Review Board (IRB) (Study number: 2019030104). The IRB stated “The IRB determined that this protocol meets the criteria for exemption from IRB review under 45 CFR 46.104 (4) Secondary research on data or specimens (no consent required).”.

### Study sample & inclusion criteria

We recruited 500 subjects through Amazon Mechanical Turk (MTurk) between June 3–8, 2020. To ensure a consistent health insurance context, participants were restricted to be U.S. residents aged 25 or older. The age restriction was imposed to exclude individuals likely covered under parental insurance plans and to ensure familiarity with personal health insurance decisions.

To maximize sample heterogeneity, the experiment was conducted in four batches at different times of day and on different days of the week. Participants were compensated $2 for completing a 10–15 minute survey. The study had a completion rate of 88.75%, indicating high data quality and reliability [7,8]. Additional details on sample size selection and experiment completion rate are provided in S6 File.

### Experiment design

**Fig 2:**
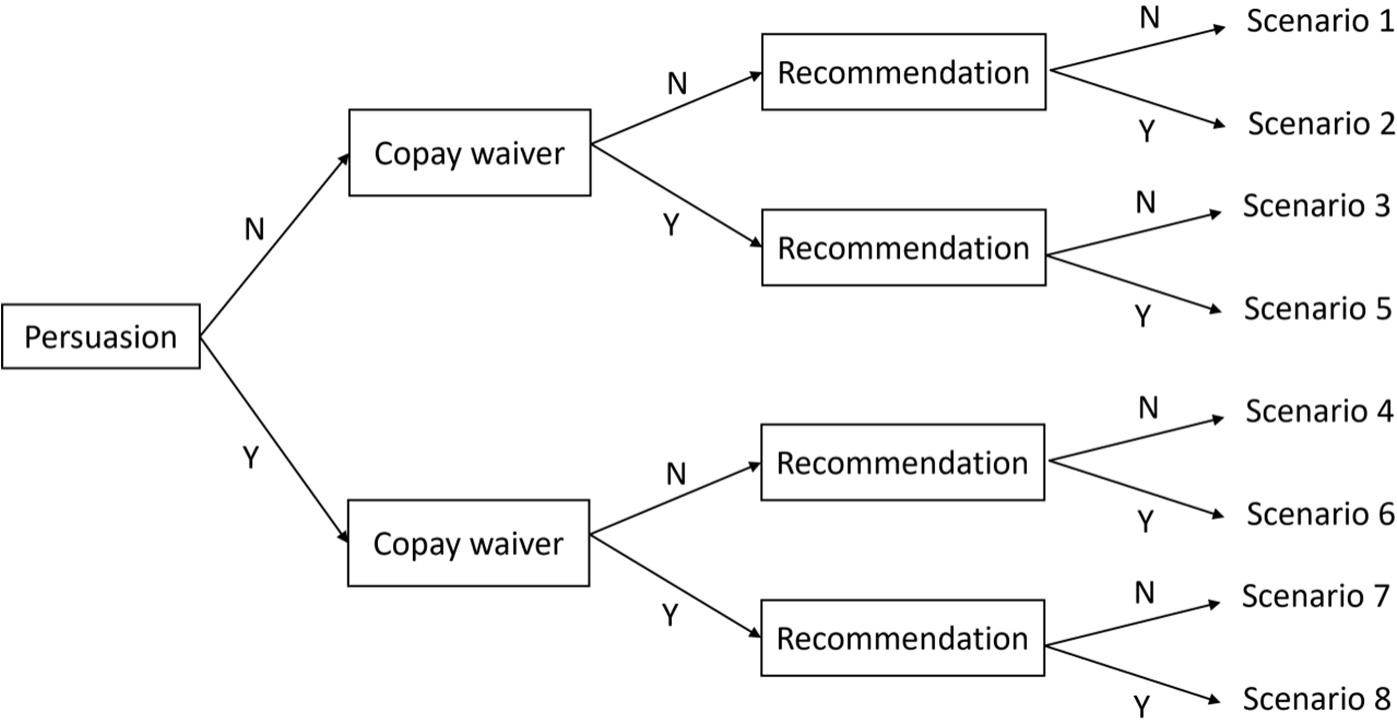
Treatments and scenarios.

We employ a full-factorial between-subjects design with three treatments: recommendation, copay waiver, and persuasion. Each treatment has two levels: 1 (Treatment = Yes) and 0 (Treatment = No), resulting in eight experimental scenarios shown in Fig 2. Subjects were randomly assigned to one scenario.

### Experimental procedures

The experiment followed a structured sequence. All survey materials and detailed question wording are provided in S5 File to facilitate replication.

1. Consent form. Subjects provided informed consent electronically prior to participating in the study. Subjects who did not provide consent were not asked to provide additional responses.
2. Background information. Subjects received a brief explanation of MRI procedures.
3. Demographics. Participants reported demographic information including gender, and categories of income, age, race, education status, employment status, English comprehension and whether subjects had insurance or not. Details on the categories of the demographic variables are provided in the survey instrument.
4. Insurance cost-sharing comprehension quiz. Subjects completed a quiz composed of one or two questions assessing their understanding of copay and coinsurance. Subjects who answered the first question correctly passed the quiz immediately. If incorrect, they were shown the correct answer and given a second question. A correct response on the second question resulted in a “Pass”; otherwise, the subject was designated as “Not Pass.” All subjects proceeded with the survey regardless of designation (Pass or Not Pass), which was not revealed to them.
5. Attention check. Subject was randomly assigned to one of four Winograd schema questions [9], which served as an attention check to identify careless respondents and assess data quality prior to analysis.
6. Scenario assignment & subjects’ provider selection decision. Subjects were randomly assigned to one of eight scenarios, which used text to present the information provided by the agent and explain the provider selection problem. Participants selected a provider based on presented information. Descriptions of the treatments and scenarios are shown in Table 1.
7. Post-scenario questions. Subjects reported reasons for their choice by answering the question “*Why did you choose X clinic?*.” In addition, they rated their level of trust in their insurance company on a 7-point Likert scale by indicating how strongly they agreed with the statement “*I trust my insurance company*,” where 1 represents strongly disagree and 7 represents strongly agree.

**Table 1:** Details of scenarios.

Sections	Text displayed to subjects [1]				
a) Background & Introduction (all scenarios)	Suppose you have a medical condition for which your doctor has recommended that you get an MRI done. Note that your condition is neither life-threatening nor urgent. Your insurance plan requires you to pay a $100 copay for the MRI. You are thinking about going to the Orange Clinic for your MRI, which is close to where you live (within 3 miles). Recently, your insurer has launched a new service: Benefits Value Advisor (BVA). If you call the BVA, you will be given information about cost and quality of providers within 3 miles’ distance from your residence.				
**b) Treatments** [2,3]					
b1) Baseline (no treatment)	You decide to try the new service and call BVA. The consultant provides you with information on four clinics below, which are all within 3 miles’ distance from your residence. Among these clinics is the Orange Clinic where you initially thought you would like to go. Your agent informs you that the four clinics have the same out-of-pocket cost to you.				
		Peach Clinic	Orange Clinic	Apple Clinic	Pear Clinic
	Price	$800	$700	$600	$500
	Ratings [4]	⋆ ⋆ ⋆ ⋆ ★	⋆ ⋆ ⋆ ⋆ ★	⋆ ⋆ ⋆ ⋆ ★	⋆ ⋆ ⋆ ⋆ ★
	Cost to you	$100	$100	$100	$100
	Cost to insurer	$700	$600	$500	$400
b2) Recommendation	Same as the baseline scenario b1), except that the following provider information was displayed. The lowest-cost provider, Pear Clinic, is labeled as the “Recommended Provider.”				
		Peach Clinic	Orange Clinic	Apple Clinic	**Pear Clinic** *[Recommended Provider]*
	Price	$800	$700	$600	**$500**
	Ratings [4]	⋆ ⋆ ⋆ ⋆ ★	⋆ ⋆ ⋆ ⋆ ★	⋆ ⋆ ⋆ ⋆ ★	⋆ ⋆ ⋆ ⋆ ★
	Cost to you	$100	$100	$100	**$100**
	Cost to insurer	$700	$600	$500	**$400**
b3) Copay Waiver	Similar to the baseline scenario b1), except that the subject saw the extra description: *After speaking to the consultant, you learn that because you tried the BVA service, your insurer decided to offer you a $100 copay waiver and your out-of-pocket cost will be $0. No matter which clinic you choose ultimately, you will get the $100 waived.* Subjects also saw the following provider information. Compared to the baseline b1), the only difference is that the patient’s out-of-pocket cost is $0 under the copay waiver treatment.				
		Peach Clinic	Orange Clinic	Apple Clinic	Pear Clinic
	Price	$800	$700	$600	$500
	Ratings [4]	⋆ ⋆ ⋆ ⋆ ★	⋆ ⋆ ⋆ ⋆ ★	⋆ ⋆ ⋆ ⋆ ★	⋆ ⋆ ⋆ ⋆ ★
	Cost to you	$0	$0	$0	$0
	Cost to insurer	$800	$700	$600	$500
b4) Persuasion by testimonial	Same as the baseline scenario b1), except that the subject saw an extra story, presented as follows [5]: *Before you make a choice, the agent shares with you an email that the program received from a beneficiary: Hi. My name is Ashley. I am a graduate student. Unfortunately, I had a meniscal tear during a basketball game two weeks ago and was required to get an MRI scan. My friend suggested that I go to some clinic, which I am going to call Clinic A. Meanwhile, I happen to learn about the BVA program, which can provide information and suggestions. I decided to call the agent for some advice and the agent provided me some options and quality information on those healthcare providers. After the conversation, I decided to choose Clinic B, which is a radiology lab that is much less expensive than Clinic A. I was apprehensive at first, but Clinic B turned out to be a great experience. They were very professional. The staff was friendly and nice. I finished my MRI scan on time. I am very satisfied with this experience.*				
**c) Provider Selection Question**					
Which provider would you choose?					
*Note:* [1] We modeled interactions during the BVA calls as scenarios with text explaining the provider selection problem and information provided by the agents. Each scenario consisted of three parts: (a) background & introduction, (b) treatment, and (c) provider selection question. Parts (a) and (c) were identical across scenarios.					
[2] Following Fig 2, the treatment descriptions for each scenario are shown below: Scenario 1 (b1), Scenario 2 (b2), Scenario 3 (b3), Scenario 4 (b4), Scenario 5 (b2 + b3), Scenario 6 (b2 + b4), Scenario 7 (b3 + b4), Scenario 8 (b2 + b3 + b4).					
[3] When both the recommendation and copay waiver treatments were present, the provider information was the same as in b3), with Pear Clinic labeled as the “Recommended Provider,” as in b2). When both the copay waiver and persuasion treatments were present, the provider information was the same as in b3), and subjects saw the testimonial in b4). When both the recommendation and persuasion treatments were present, Pear Clinic was labeled as the “Recommended Provider,” as in b2), and subjects saw the testimonial in b4).					
[4] The quality ratings were set at 4.5 out of 5 for all providers and held constant across all providers and scenarios.					
[5] This testimonial is a fictional story created solely to simulate the testimonial-based persuasion used in the BVA program. It does not reflect any real beneficiary’s experience, and no actual patient or beneficiary information was used.					

The interactions between agents and beneficiaries were modeled as text-based scenarios describing the provider selection problem and information provided by agents. The scenarios were based on the actual setting of the BVA program, with the patient’s out-of-pocket for the MRI set at $100. MRI procedures were chosen as they were the shoppable medical services targeted by the BVA program because of large variation in provider costs for these procedures.

Additionally, neutral names (Peach, Orange, Apple and Pear Clinic) were used for service providers to ensure that subjects would neither recognize nor associate provider names with service quality. The four clinics differed only in price, which followed the order: Peach Clinic > Orange Clinic > Apple Clinic > Pear Clinic. A five-star rating system was used to capture the service quality, as it is the most widely adopted format for evaluating healthcare providers. Quality ratings were held constant across all providers, as the BCBS web site had many providers with nearly identical five-star ratings despite substantial price differences. The provider names, prices, and quality ratings did not vary across all eight scenarios. In addition, the requested provider was set as a relatively high-cost provider (i.e., Orange Clinic, the second highest provider), which was common in BCBS data and consistent with the pattern that high cost providers tend to have greater market visibility.

### Dependent measures

The analysis focuses on two binary measures: 1) whether subjects chose the lowest-cost provider, i.e., Pear Clinic, and 2) whether subjects chose a provider that was lower-cost than the requested provider, i.e., whether it was either Apple or Pear Clinic. The second measure reflects the real-world success metric used by BCBS, where any cost reduction relative to beneficiaries’ initial requests is deemed a success. The first measure is aligned with the recommendation treatment, which explicitly guides patients toward the best-value option. Note that the Pear Clinic is the best-value option given the identical quality ratings across all four providers. We refer to the first measure as M1 and the second as M2.

### Descriptive statistical analysis

The descriptive analysis employed unpaired sample t-tests, χ2 tests, and Fisher’s exact tests. To ensure a balanced sample across the eight scenarios, χ2 tests were conducted for each demographic variable. Next, provider selection outcomes were compared across treatment levels to descriptively examine the impact of treatments on the proportions of subjects selecting either the lowest-cost provider or any lower-cost provider. The analysis also measured subjects’ trust in their insurance companies using their responses to the statement, *“I trust my insurance company.”* Based on these responses, subjects were categorized into two groups: (1) Mistrust (i.e., Strongly disagree, Disagree, Somewhat disagree), and (2) Do Not Mistrust (i.e., Neither agree or disagree, Somewhat agree, Agree, Strongly agree). Using this classification, we examined whether mistrust moderates the treatment effect by analyzing the association between recommendations and the selection of a lowest- or lower-cost provider. Additionally, we analyzed the association between subjects’ understanding of cost-sharing terms (as measured by quiz performance) and their provider selection.

### Statistical methods

The primary statistical analysis tool employed is logit regression. A logit specification is common in settings where the dependent variable is binary. The analysis begins with a preliminary model examining the overall effects of the three treatments. The specification is shown in Eq (1). All regression variables are defined in Table 2. Coefficients $\beta_{1}$, $\beta_{2}$ and $\beta_{3}$ correspond to the treatments: recommendation, copay waiver and persuasion by testimonial, respectively. Parameter $\beta^{C}$ is a vector of coefficients associated with control variables **C**_**i**_, which includes subject *i*’s gender, insurance status, income, age, race, education, employment status, and English proficiency.


$$\begin{array}{cc} \text{logit}(P_{i})\;\;\;= & \beta_{0}+\beta_{1}{\text{Recommendation}}_{i}+\beta_{2}{\text{CopayWaiver}}_{i}+\beta_{3}{\text{Persuasion}}_{i}\\ & +\beta^{C}C_{i}+\epsilon_{i}. \end{array}$$ (1)

**Table 2:** Definitions of variables used in the regression analysis.

Variables name	Definition
*i*	Subject ID
$ChoosePear_{i}\in \left\{0,1\right\}$	M1: Whether subject *i* chooses the lowest-cost provider (Pear Clinic)
$ChooseLower_{i}\in \left\{0,1\right\}$	M2: Whether subject *i* chooses the lower-cost providers (Pear/Apple Clinic)
$P_{i}$	Probability that the binary dependent measure equals 1 for subject *i*.
	If M1 is used, then $P_{i}=\text{Prob}({\text{ChoosePear}}_{i}=1)$.
	If M2 is used, then $P_{i}=\text{Prob}({\text{ChooseLower}}_{i}=1)$
$Recommendation_{i}\in \left\{0,1\right\}$	1 indicates recommendation treatment for subject *i*
$CopayWaiver_{i}\in \left\{0,1\right\}$	1 indicates copay waiver treatment for subject *i*
$Persuasion_{i}\in \left\{0,1\right\}$	1 indicates persuasion by testimonial treatment for subject *i*
$Mistrust_{i}\in \left\{Mistrust,Do\;Not\;Mistrust\right\}$	Subject *i*’s mistrust towards the insurance company.
	$Mistrust_{i}=1$ if subject *i* chooses *Strongly disagree/Disagree/Somewhat disagree*
	$Mistrust_{i}=0$ if subject *i* chooses *Neither agree or disagree/*
	*Somewhat agree/Agree/Strongly agree.*
$Pass_{i}\in \left\{0,1\right\}$	Whether subject *i* passes the quiz on insurance cost-sharing terms
$X_{i}$	A vector of between-treatment interactions for subject *i*
**C**_**i**_	A vector of all control variables for subject *i*, which includes eight demographic variables

Next, the model in Eq (2) is fitted to the data. This model incorporates all interaction terms. Coefficients $\beta_{4}$ and $\beta_{5}$ are associated with the subject’s mistrust level and whether they pass the quiz on the meaning of health insurance cost-sharing terms (i.e., co-pay and co-insurance). Parameters $\beta_{6}$, $\beta_{7}$ and $\beta_{8}$ are the coefficients for the interaction terms between mistrust level and the three treatments, respectively. For subject *i*, the vector $X_{i}=(X_{i,1},X_{i,2},X_{i,3})$ includes the between-treatment interaction terms, i.e., $X_{i,1}={\text{Recommendation}}_{i}{\text{CopayWaiver}}_{i}$, $X_{i,2}={\text{Recommendation}}_{i}{\text{Persuasion}}_{i}$, and $X_{i,3}={\text{CopayWaiver}}_{i}{\text{Persuasion}}_{i}$. The vector $\beta^{I}$ is a vector of coefficients associated with interaction terms in $X_{i}$.


$$\begin{array}{cc} \text{logit}(P_{i})\;\;\;= & \beta_{0}+\beta_{1}{\text{Recommendation}}_{i}+\beta_{2}{\text{CopayWaiver}}_{i}+\beta_{3}{\text{Persuasion}}_{i}\\ & +\beta_{4}{\text{Mistrust}}_{i}+\beta_{5}{\text{Pass}}_{i}+\beta_{6}{\text{Recommendation}}_{i}{\text{Mistrust}}_{i}\\ & +\beta_{7}{\text{CopayWaiver}}_{i}{\text{Mistrust}}_{i}+\beta_{8}{\text{Persuasion}}_{i}{\text{Mistrust}}_{i}\\ & +\beta^{I}X_{i}+\beta^{C}C_{i}+\epsilon_{i}. \end{array}$$ (2)

The inclusion of subjects’ mistrust level and their quiz performance in the comprehension of co-pay and co-insurance is based on the observed correlation between these variables and provider selection decision (see Descriptive statistics).

### Robustness checks

Four robustness checks are implemented to validate the findings. The first analysis uses results of the Winograd questions. The primary analysis does not screen out the respondents who fail the Winograd attention check, thus two additional analyses are performed as a robustness check: (1) fitting the model only to responses from subjects who pass the attention check, and (2) including a control variable indicating whether a subject passes the attention check. Second, to address the issue of imbalanced data caused by much fewer respondents mistrusting their insurance company, an additional analysis employs an oversampling approach via bootstrapping. To mitigate overfitting concerns in this exercise, the minority class (respondents expressing mistrust) is progressively oversampled at twice, three times, four times, and five times its original size, with the regression models re-estimated at each increment. Third, a counterfactual analysis compares the experimental results and the claims data in aggregate (see S12 File). We estimate a multinomial logit model using the experimental data, with provider choice among the four providers as the dependent variable and the same set of covariates as in Eq (2). We then use the estimated model to predict the proportion of subjects selecting lower-cost providers and compare these predictions with the actual proportion of beneficiaries choosing lower-cost providers observed in the claims data. Lastly, in addition to the logit specification, we also estimate probit specification of Eqs (1) and (2), which are otherwise identically specified.

## Results

### Descriptive statistics

**Demographic profiles** Two observations from subjects with the same IP address were dropped, resulting in 498 observations for analysis. The number of observations for Scenarios 1–8 were 58, 59, 65, 63, 62, 69, 60, and 62, respectively. In accordance with ethical guidelines, we report only aggregated summary statistics for the demographic variables. Among the subjects, 50.6% were female, 81.5% were white, and 86.94% were aged 25–54. Additionally, 78.9% were employed full-time or part-time, 86% had healthcare insurance, and 65.7% held a bachelor’s degree or higher. The demographic profile of the online sample aligned with other reported survey samples from MTurk [10], which were younger and more educated than national average.

The $\chi^{2}$ tests were performed for each demographic variable across the eight scenarios. The results showed no significant differences in subjects’ demographic characteristics, confirming homogeneous subject populations across the eight scenarios.

**Subjects’ provider selection** Provider selection results are summarized in Table 3. We focus on two measures: (i) the proportion of subjects choosing the lowest-cost provider (Pear Clinic, M1), and (ii) the proportion choosing lower-cost providers (Apple or Pear Clinic, M2).

**Table 3:** Summary of subjects’ responses.

		Provider Selection (X Clinic)				
Scenarios	Treatments	Peach	Orange	Apple	Pear	Apple or Pear
					(Lowest-cost)	(Lower-cost)
1	Null	10 (17.24%)	26 (44.83%)	4 (6.90%)	18 (31.03%)	22 (37.93%)
2	Recommendation	6 (10.17%)	20 (33.90%)	0 (0.00%)	33 (55.93%)	33 (55.93%)
3	Copay Waiver	15 (23.08%)	33 (50.77%)	3 (4.62%)	14 (21.54%)	17 (26.16%)
4	Persuasion	10 (15.87%)	33 (52.38%)	5 (7.94%)	15 (23.81%)	20 (31.75%)
5	Recommendation + Copay Waiver	4 (6.45%)	19 (30.65%)	3 (4.84%)	36 (58.06%)	39 (62.90%)
6	Recommendation + Persuasion	9 (13.04%)	27 (39.13%)	2 (2.90%)	31 (44.93%)	33 (47.83%)
7	Copay Waiver + Persuasion	10 (16.67%)	32 (53.33%)	5 (8.33%)	13 (21.67%)	18 (30.00%)
8	Recommendation + Copay Waiver + Persuasion	5 (8.06%)	25 (40.32%)	1 (1.61%)	31 (50.00%)	32 (51.61%)
Total		69 (13.86%)	215 (43.17%)	23 (4.62%)	191 (38.35%)	214 (42.97%)

Notes: Numbers in the first row show the frequency and numbers in the second row indicate the percentage of subjects choosing a specific clinic in each scenario (i.e., row percentage).

The descriptive results reveal several patterns. First, recommendation has a strong positive effect on the choice of the lowest-cost provider. Relative to the control group (Scenario 1), the proportion of subjects selecting the lowest-cost provider increases substantially when there is a recommendation. A similar pattern is observed for M2. In contrast, neither copay waiver nor persuasion by itself appears to meaningfully influence either measure. In both cases, the proportion of subjects selecting the lowest-cost provider or lower-cost options is lower than that in the control group.

**Level of mistrust** Table 4 summarizes the subjects’ attitude towards their insurance companies. Among all subjects, 15.66% had a negative attitude towards their insurance company in terms of trust level (i.e., they responded Strongly disagree, Disagree, Somewhat disagree to the question concerning trust level). After reclassifying subjects’ trust level into two groups (i.e., Mistrust and Do Not Mistrust), it was found that the effect of recommendation on steering subjects’ choice to the lowest-cost provider (and lower-cost providers) was related to their mistrust level. Table 5 illustrates the association between recommendation and the count of subjects who choose the lowest-cost provider (and lower-cost providers) under each mistrust level. The $\chi^{2}$ and Fisher’s exact tests were conducted to examine whether there were differences in two dependent measures under recommendation versus no recommendation. The results showed that while there was no association between the differences in dependent measures for subjects who mistrusted their insurance companies, there was a significant correlation for those who did not mistrust their insurance company. This suggests that mistrust may be an important moderator of subjects’ choice behavior.

**Table 4:** Subjects’ response to the statement *“I trust my insurance company”.*

Responses	Frequency (Percentage)	Re-categorization	
Strongly disagree	14 (2.81%)	Mistrust	15.66%
Disagree	18 (3.61%)		
Somewhat disagree	46 (9.24%)		
Neither agree or disagree	73 (14.66%)	Do Not Mistrust	84.34%
Somewhat agree	141 (28.31%)		
Agree	144 (28.92%)		
Strongly agree	62 (12.45%)		

**Table 5:** Heterogeneity of recommendations effect across subject subgroups with different mistrust levels in insurance companies.

**M1:** Proportion of subjects choosing the lowest-cost provider			
	No recommendation	With recommendation	p−value
Mistrust	28.57%	32.56%	$\chi^{2}$ test: *p* = 0.704; Fisher exact test: *p* = 0.807
Do Not Mistrust	23.70%	55.98%	$\chi^{2}$ test: *p* < 0.01; Fisher exact test: *p* < 0.01
**M2:** Proportion of subjects choosing the lower-cost provider			
	No recommendation	With recommendation	p−value
Mistrust	34.29%	34.88%	$\chi^{2}$ test: *p* = 0.956; Fisher exact test: *p* = 1.000
Do Not Mistrust	30.81%	58.37%	$\chi^{2}$ test: *p* < 0.01; Fisher exact test: *p* < 0.01

Notes: “No Recommendation” refers to scenarios without the recommendation treatment (i.e., Scenarios 1,3,4,7), while “With Recommendation” refers to scenarios with the recommendation treatment (i.e., Scenarios 2,5,6,8).

**Understanding of insurance cost sharing** In the study cohort, 80.92% of the subjects passed the quiz (see Table 6, part (1)). The analysis categorized those subjects who passed the quiz as “Yes” in the Table 6, part (2), and the rest as “No,” regardless of their treatment assignments. There was a strong correlation between subjects’ quiz performance and their provider choices (see Table 6, part (2)). Additionally, passing the quiz was strongly associated with choosing the lowest-cost provider and lower-cost providers (see Table 6, parts (3) and (4)). This suggested that subjects with a good understanding of the cost sharing were more likely to choose the lowest-cost provider and lower-cost providers.

**Table 6:** Description of quiz performance and its correlation with provider choice.

(1) Good understanding					
Pass the quiz?	Frequency		Percent		
Yes	403		80.92%		
No	95		19.08%		
(2) Provider choice					
Pass the quiz?	Peach Clinic	Orange Clinic	Apple Clinic	Pear Clinic	
Yes	48 (11.91%)	167 (41.44%)	15 (3.72%)	173 (42.93%)	Pearson chi2(3): *p* < 0.01;
No	21 (22.11%)	48 (50.53%)	8 (8.42%)	18 (18.95%)	Fisher’s exact: *p* < 0.01
In total	69 (13.86%)	215 (43.17%)	23 (4.62%)	191 (38.35%)	
(3) Choice of the lowest-cost provider (M1)					
Pass the quiz?	Yes		No		
Yes	173 (42.93%)		230 (57.07%)		Pearson chi2(1): *p* < 0.001;
No	18 (18.95%)		77 (81.05%)		Fisher’s exact: *p* < 0.001
In total	191 (38.35%)		307 (61.65%)		
(4) Choice of the lower-cost provider (M2)					
Pass the quiz?	Yes		No		
Yes	188 (42.97%)		215 (57.03%)		Pearson chi2(1): *p* = 0.001;
No	26 (57.03%)		69 (72.63%)		Fisher’s exact: *p* = 0.001
In total	214 (42.97%)		284 (57.03%)		

### Results of regression analysis

**Preliminary results** Regression results for Model (1) are summarized in Table 7. The results from an approach in which control variables are added sequentially are presented in S7 File. The reported coefficients represent changes in the log-odds of the outcomes (i.e., M1, M2) associated with a one-unit change in the treatment, which are not directly interpretable in probability terms. To facilitate interpretation, we report Average Marginal Effects (AMEs), which reflects the average change in the probability of subjects choosing the lowest-cost provider (or lower-cost providers) when the treatment changes from 0 to 1, holding other variables constant. The results confirm the earlier finding that recommendation has a positive impact on subjects choosing the lowest-cost provider and lower-cost providers, but copay waiver and persuasion do not have a significant impact on either measure. The AME indicates that the recommendation treatment increases the probability of subjects choosing the lowest-cost provider by 28.6% and the probability of selecting lower-cost providers by 23.5%.

**Table 7:** Regression results for the preliminary model.

**M1: Choosing Lowest-cost Provider**	With Controls	
	Coefficient	AME
**Recommendation**	−1.333^***^	−0.286^***^
**CopayWaiver**	−0.141	−0.029
**Persuasion**	−0.321	−0.066
Control Variables	Yes†	
Observations	482‡	
Pseudo *R*^2^	0.1084	
**M2: Choosing Lower-cost Providers**	With Controls	
	Coefficient	AME
**Recommendation**	−1.037^***^	−0.235^***^
**CopayWaiver**	−0.110	−0.024
**Persuasion**	−0.239	−0.052
Control Variables	Yes†	
Observations	482‡	
Pseudo *R*^2^	0.0829	

Notes: *** *p* < 0.01, ** *p* < 0.05, * *p* < 0.1. AME stands for Average Marginal Effect.

† Control variables: Gender, Insurance, Income, Age, Race, Education, Employment Status, English Proficiency.

‡ Sixteen observations were dropped for the following reason. Certain categories within the demographic variables contained a small number of observations. When all subjects within such a category selected the same provider, this resulted in model fit failure, and those observations were excluded.

**Comprehensive regression analysis results** The results for the comprehensive model are presented in Table 8, displaying only the significant interactions. The complete results including all interactions can be found in S8 File.

**Table 8:** Regression Results for Comprehensive Model.

M1: Choosing Lowest-Cost Provider	With Controls	
	Coefficient	AME
**Recommendation**	−1.506^***^	−0.327^***^
**CopayWaiver**	−0.621	−0.111
**Persuasion**	−0.407	−0.076
**Mistrust**	−0.013	−0.003
**Recommendation + Mistrust**		
0 *Do Not Mistrust (base level)*		
1 *Mistrust*	−1.454^***^	−0.274^***^
**Pass**	−1.034^***^	−0.188^***^
Control Variables	Yes†	
Observations	482‡	
Pseudo *R*^2^	0.1485	
**M2: Choosing Lower-Cost Providers**	**With Controls**	
	**Coefficient**	**AME**
**Recommendation**	−1.211^***^	−0.273^***^
**CopayWaiver**	−0.462	−0.094
**Persuasion**	−0.161	−0.034
**Mistrust**	−0.226	−0.050
**Recommendation + Mistrust**		
0 *Do Not Mistrust (base level)*		
1 *Mistrust*	−1.387^**^	−0.276^***^
**Pass**	−0.599^**^	−0.124^**^
Control Variables	Yes†	
Observations	482‡	
Pseudo *R*^2^	0.1100	

Notes: *** *p* < 0.01, ** *p* < 0.05, * *p* < 0.1.

† Control variables: Gender, Insurance, Income, Age, Race, Education, Employment Status, English Proficiency

The CopayWaiver + Mistrust, Persuasion + Mistrust are not shown here because these two interactions are not significant. Full results can be found in S8 File.

‡ Sixteen observations were dropped for the following reason. Certain categories within the demographic variables contained a small number of observations. When all subjects within such a category selected the same provider, this resulted in model fit failure, and those observations were excluded.

The findings are as follows. First, recommendation has a positive impact on subjects’ choice of the lowest-cost provider. Looking at AMEs, subjects receiving recommendations are predicted to have 32.7% higher likelihood of selecting the lowest-cost provider and 27.3% higher chance of choosing lower-cost providers. Second, the recommendation effect decreases when subjects mistrust their insurance companies. When receiving recommendations, subjects who mistrust their insurance company are predicted to be 27.4% less likely to choose the lowest-cost one and 27.6% less likely to choose lower-cost providers. However, even with mistrust, recommendation maintains a net positive effect on choosing the lowest-cost provider, though not for selecting lower-cost providers (i.e., 32.7%−27.4% = 5.3% for M1; 27.3%−27.6% = −0.3% for M2). Third, copay and persuasion do not have a significant impact on the choice of either the lowest-cost providers or lower-cost providers. Lastly, whether subjects pass the quiz pertaining to the knowledge of health insurance cost-sharing has a significant impact on both the choice of the lowest-cost provider and lower-cost providers. Subjects who pass the quiz are predicted to be 18.8% more likely to choose the lowest-cost provider and 12.4% more likely to select lower-cost providers, holding all else constant.

While our models explain a modest portion of the total variance in the outcome (Pseudo *R*^2^ ranges from 8.29% to 14.85% in Tables 7 and 8), the primary focus of this study is on the consistent and theoretically-grounded relationship between the three treatments and two dependent measures. The low R-squared is common in studies involving human behavior and decision making, as a great deal of the variation is inherently idiosyncratic and unobservable. We provide further investigation on the subjects’ provider choices in the Discussion section and S13 File. The significant coefficients and AMEs nonetheless provide valuable insights into the directional effect and relative importance of these factors.

### Robustness check results

First, among all subjects, 79.52% passed the Winograd check and the remaining 20.48% failed. Subjects were more likely to have a good understanding of the cost sharing if they passed the attention check (see S10 File) although the Winograd questions had nothing to do with provider choice whatsoever. The two additional analyses, mentioned earlier, produced results similar to the original analysis (see S10 File). That is, only recommendation significantly increased the likelihood of subjects selecting lowest- and lower-cost providers and the effect was undermined if subjects mistrusted the insurance company. Second, through the oversampling, it was found that the key results remain qualitatively consistent throughout (see S11 File). Again, recommendation was the only effective strategy in steering subjects’ choice to lowest- and lower-cost providers, with its effect moderated by the level of mistrust. Third, the counterfactual analysis showed that the results were consistent with observed claims data in the aggregate (see S12 File). Recall that we compared the estimated proportion of subjects choosing lower-cost providers with the actual proportion of beneficiaries choosing a lower-cost provider in the claims data. The predicted proportion of subjects choosing a lower-cost provider was 53.2%, closely matching the 53% observed in claims data. Note that this consistency should be interpreted as supportive, rather than conclusive, evidence for the validity of our findings, as the study remains subject to the behavioral limitations outlined in the Behavioral Limitations & External Validity of the Discussion section. Lastly, we fitted the probit version of both the preliminary and comprehensive regression models. All the results remained consistent (see S9 File).

## Discussion

### Summary of findings

Our experiment sheds light on the four research questions we highlighted in the Introduction section. A summary of these findings is presented below.

1. In response to the question “what proportion of beneficiaries change their initial choice of provider?,” we find that without any intervention (i.e., Scenario 1 in Table 3), 31.03% subjects chose the lowest-cost provider and 37.93% chose one of the lower-cost providers.
2. In response to the question “Which strategy is most effective in increasing the proportion of beneficiaries switching to lower-cost providers?,” we find that recommendation affects subjects’ choices the most. Subjects receiving recommendations are predicted to have 32.7% higher likelihood of selecting the lowest-cost provider and 27.3% higher chance of choosing lower-cost providers. Moreover, copay waiver and testimonials do not significantly affect beneficiaries’ choices.
3. In response to the question “Which individual characteristics have the greatest impact on beneficiaries choices?,” we find that mistrust and comprehension of cost sharing concepts, i.e., copay and coinsurance, are the highest impact factors. Mistrust undermines the recommendation effect, whereas greater comprehension of cost sharing increases the likelihood that a subject will choose the lowest-cost provider and lower-cost providers.
4. First, the findings suggest that recommendation may be more effective than copay waivers or testimonial persuasion in influencing provider choice. Second, the results highlight the potential importance of reducing mistrust and increasing beneficiaries’ comprehension of cost-sharing. Improving transparency around how provider choices affect both individual out-of-pocket expenses and broader insurance costs may further improve the effectiveness of such interventions.

We next utilize insights we gleaned from the literature to explain the theoretical underpinnings of our findings.

**Possible mechanisms** The impact of word-of-mouth (WOM) recommendations on consumer decision-making is well-documented, with effectiveness influenced by social ties and perceived risk [11,12]. Social ties refer to interactions between individuals [13], and tie strength reflects the closeness of these relationships [14]. Strong ties, such as family and close friends, are typically more influential than weak ties, which include strangers or casual acquaintances [11]. In this context, an agent’s recommendation resembles a WOM recommendation with weak social ties. Consumers often prefer weak-tie sources for technical products requiring specialized knowledge and with higher perceived risks [12,15]. Health care products are complex with higher perceived outcome risks and information asymmetry, which drives beneficiaries to seek recommendations from various sources to assess provider quality and avoid service failures [12,16]. An agent’s recommendation can serve as expert guidance, aiding their decision-making process. This explains the effectiveness of the recommendation treatment in our experiment. Meanwhile, people scrutinize source trustworthiness more closely in the recommendation seeking process when perceived risks are high [15]. This aligns with our finding that the credibility of the agent’s recommendation diminishes when subjects mistrust their insurance company.

Focusing next on the lack of effectiveness of copay waiver, we first postulate that the insurer’s expectation that a copay waiver may be effective is rooted in the reciprocity effect which refers to the tendency to respond to others’ intentions by rewarding kindness and punishing unkindness [17–20]. That is, in our experiment the unconditional copay waiver is designed to be a gesture of goodwill from the insurer. However, this backfired. Drawn from the experiment’s results, we conjecture that subjects may interpret the waiver not as genuine kindness but as a strategic maneuver to direct them toward lower-quality providers. The potential suspicion of insurer’s intention could explain the lack of the reciprocity effect.

The testimonial effect is tied to persuasion, defined as “human communication designed to influence the judgments and actions of others” [21]. Unlike inducement or coercion, persuasion does not restrict options, or significantly alter economic incentives, or impose penalties. Its effectiveness depends on three key factors: persuasion knowledge (how individuals cope with persuasion), agent knowledge (beliefs about the persuader’s traits, tactics, and goals), and topic familiarity (prior experience with the subject) [22]. Similar to copay waiver, we conjecture that the suspicion of the insurer’s motives undermines the subjects’ perception of agents’ knowledge, which explains the lack of effectiveness of persuasion treatment in the experiment.

A limitation of our experiment in this context is that we did not test other testimonials. While sharing patient experiences in one-on-one beneficiary-agent interactions seem to be ineffective, other approaches remain unexplored and may produce positive outcomes.

A possible explanation for the association between a better understanding of the cost sharing and a greater propensity to choose the lowest-cost providers or lower-cost providers is that subjects who understand their costs are more likely to recognize the connection between their choices and the long-term cost of health services (see S13 File). Therefore, they are more inclined to choose a low-cost provider as long as the quality is not compromised.

### Behavioral limitations & external validity

While the experimental design allows for controlled identification of treatment effects, subjects’ choices in this hypothetical setting may differ from real-world beneficiary behavior in several important ways.

First, the decision context is simplified. In practice, provider choice is influenced by additional factors such as physician referrals, appointment availability, provider capacity, and urgency of medical needs. These constraints are intentionally abstracted away in our design to isolate the causal impact of the three strategies, but may limit behavioral realism. In addition, while we limit the providers’ location to within 3 miles distance from a participant’ residence, individuals in real settings may prefer the most conveniently located provider.

Second, the experiment does not fully capture physician influence. While our design uses the requested provider to approximate potential physician recommendations, in practice such influence often takes the form of physician referrals, which exerts a stronger effect and could be a driver of provider selection.

Third, subjects’ responses may be subject to hypothetical bias [23]. Because choices are not tied to real consequences, participants may behave differently than they would in practice. For example, some individuals may select lower-cost providers to signal altruism and present themselves as “good Samaritans,” even if they would prioritize other factors such as convenience or appointment availability in real decision contexts.

While our counterfactual analysis suggests that the experimental patterns are broadly consistent with observed aggregate claims data (see S11 File), still, the results should be interpreted as providing suggestive evidence rather than directly generalizable estimates of real-world behavior because of the aforementioned limitations. Future research using field data or real-world interventions would be valuable to further assess their external validity.

### Qualitative analysis of the underlying reasons for subjects’ choices

The survey also asked subjects to report their reasons for selecting a particular provider after making their final decision. Their responses highlighted the multitude of motivations that influence beneficiaries choice of lowest-cost and lower-cost providers. Details of the qualitative analysis of these self-reported reasons are provided in S13 File. We summarize the main observations here.

- Subjects care about the value delivered by providers, and are willing to select lower-cost options among those with similar quality. Among the 191 subjects who selected Pear Clinic (lowest-cost provider) across eight scenarios, 59% mentioned it as the best-value provider.
- A significant proportion of subjects exhibit altruism by selecting options that reduce costs for health insurance companies. For instance, 44% chose Pear Clinic to save costs for their insurer.
- Many subjects tend to stick with their initial choice. Among the 215 subjects who selected Orange Clinic, the second-highest priced one and the requested provider, 63% cited that they preferred to stick to their initial choice.
- A substantial number of subjects believe in a positive correlation between provider prices and quality, leading them to choose expensive options. For example, 5% of subjects who chose Orange Clinic believed it had higher quality. Meanwhile, among the 69 subjects selecting Peach Clinic, the highest-cost provider, 86% believed it had the best quality.

### Other payment innovations

In addition to BVA-like health advocacy programs, insurers have utilized other approaches to help lower costs and improve quality [24,25]. For example, Reference Pricing (RP) and Rewards Program are two alternative approaches that steer beneficiary choices to low-cost providers. In the RP scheme, the insurer sets the maximum amount, called reference price, that can be reimbursed for a procedure. If a patient selects a provider that charges more than the reference price, then the patient is responsible for the portion in excess of the reference price in addition to the copayment or the coinsurance portion of the reference price, as applicable. The Rewards Program pays a monetary reward to beneficiaries who choose a low-cost provider [24]. Unlike the RP Scheme and the Rewards Program, beneficiaries’ choices do not directly influence their out-of-pocket costs under the BVA program. This characteristic retains the moral hazard inherent in health insurance services, making the program unique. Therefore, the insights do not directly apply to RP and Rewards Program. More details on the healthcare payment innovations are provided in S14 File.

### Use of MTurk

MTurk was chosen for several reasons. First, an experimental approach is necessary to establish a causal relationship. Even if observational data were available, numerous confounding factors, such as variations in interactions between agents and beneficiaries, as well as the tone and manner of communication would be difficult to control for. Our text-based experimental design allows for control over these variables. Furthermore, due to ethical concerns, IRB typically prohibit such experiments on real patients, leading researchers to conduct similar studies in behavioral labs with university students. However, college students are typically under 25 and lack the demographic and socioeconomic diversity that is necessary for this study. Lastly, although generative AI could be used to mimic human-like responses to questions pertaining to preference, such experiments do not replicate real participants’ responses [26]. Therefore, directly using large language models in place of human respondents for preference elicitation may be misleading [27]. For these reasons we chose MTurk, which has been widely used in health services, psychology, political science, and marketing research, consistently providing representative and reliable samples [28–33]. Additional justification for the choice of MTurk for conducting the experiment is presented in S15 File.

### Other limitations

Besides limitations in external validity, our study has several other limitations. First, in terms of the experiment format, we only utilized text-based scenarios to avoid the confounding factors inherent in other formats (e.g., accent and tone in audio format, and identification with the narrator’s race and skin color in the video format). Additionally, we did not test all forms of testimonial and did not vary the copayment amount. These would be the avenues for future research. Lastly, the sample obtained from the MTurk is younger than national population. Future studies may include more elderly subjects to improve the generalizability.

## Supporting information

S1 Fig (1) Requested versus Recommended amount. (2) Percentage of times providers belonged to different price quartile by requested versus recommended. The price quartile is determined based on requested amount. (TIF)

S2 Fig An example of MRI Scan. Computer-generated image by the authors using Claude. Note that the figure is similar but not identical to the image we used in the survey and is therefore for illustrative purposes only.
(TIF)

S3 Fig Provider information. (TIF)

S4 Fig Provider information with recommendation. (TIF)

S5 Fig Provider information with copay waiver. (TIF)

S6 Fig Provider information with copay waiver & recommendation. (TIF)

S7 Fig Top reasons for choosing pear & orange clinic. (TIF)

S1 File S1 Appendix. Similar health advocate programs.
(PDF)

S2 File S2 Appendix. Preliminary Analysis of BCBS Claims Data.
(PDF)

S3 File S3 Appendix. Payment structure in the BVA program.
(PDF)

S4 File S4 Appendix. Price negotiations for non-urgent and shoppable procedures & misperception of price-quality relationship.
(PDF)

S5 File S5 Appendix. The survey instrument.
(PDF)

S6 File S6 Appendix. Sample size selection & mturk experiment completion rate.
(PDF)

S7 File S7 Appendix. Results of Preliminary model – logit regression.
(PDF)

S8 File S8 Appendix. Results of comprehensive model – logit regression.
(PDF)

S9 File S9 Appendix. Results of preliminary and comprehensive models – probit regression.
(PDF)

S10 File S10 Appendix. Additional analysis involving winograd Check.
(PDF)

S11 File S11 Appendix. Oversampling results.
(PDF)

S12 File S12 Appendix. External validity.
(PDF)

S13 File S13 Appendix. Qualitative analysis of the underlying reasons for subjects’ choices.
(PDF)

S14 File S14 Appendix. Healthcare payment innovations.
(PDF)

S15 File S15 Appendix. Use of mechanical turk.
(PDF)
