# Supplementary material for: An evaluation of strategies commonly used by health advocate programs
Source: PLoS One. 2026 Jul 17;21(7):e0350645. doi: 10.1371/journal.pone.0350645 (PMC13379028; doi:10.1371/journal.pone.0350645)
Supplement: S1 File — Similar health advocate programs. (PDF) [file pone.0350645.s007.pdf]

S1 Appendix Similar Health Advocate Programs

Program	Organization	Key Features
Advocate4Me	UnitedHealthcare	Single contact; cost/quality comparison; claims support https://www.uhc.com/health-and-wellness/member-resources/advocate4me
Health Concierge	Aetna	Personalized guidance; provider search; billing help https://www.aetna.com/employers-organizations/aetna-health-concierge.html
One Guide	Cigna	Plan selection; provider/cost tools; claims assistance https://www.cigna.com/individuals-families/member-resources/one-guide
Member Services	Humana	Benefits help; provider steering; clinical coordination https://www.humana.com/employer
Care Navigation	Kaiser Permanente	Appointment support; coverage/cost guidance; digital tools https://healthy.kaiserpermanente.org/support
Navigate4Me	Optum	Dedicated guide; complex care coordination https://www.optum.com/business/solutions/population-health/navigate4me.html
Accolade	Vendor	Concierge advocacy; clinical integration; claims resolution https://www.accolade.com/
Quantum Health	Vendor	Real-time intercepts; provider coordination; PA support https://quantum-health.com/
Included Health	Vendor	Care navigation; specialty guidance; virtual care https://includedhealth.com/solutions/care-navigation/
Rightway	Vendor	Health guides; cost transparency; pharmacy support https://www.rightwayhealthcare.com/
Health Advocate	Vendor	Claims/billing resolution; provider search; coordination https://www.healthadvocate.com/
Castlight (Vera)	Vendor	Digital navigation; cost tools; primary care integration https://www.castlighthealth.com/

Table 9. Health Advocate Programs Similar to the BVA Program
