# Supplementary material for: An evaluation of strategies commonly used by health advocate programs
Source: PLoS One. 2026 Jul 17;21(7):e0350645. doi: 10.1371/journal.pone.0350645 (PMC13379028; doi:10.1371/journal.pone.0350645)
Supplement: S2 File — Preliminary Analysis of BCBS Claims Data. (PDF) [file pone.0350645.s008.pdf]

S2 Appendix. Preliminary Analysis of BCBS Claims Data The analysis utilizes BCBS claims data containing call records from nine Texas cities during 2014-2017. The data contain 40,137 records in total. Each record represents a call to an agent. It contains the date of call, the location of the beneficiary, the procedure being requested, the ID of the serving agent, the requested provider, the recommended provider and the actual payment amount. Data also include costs of both the requested provider and the recommended provider. There are 20 procedure groups, including common procedures MRI, CT Scan, and joint replacement. The focus of the study is on shoppable diagnostic procedures, specifically MRI and CT Scan procedures, which account for 32,483 (81%) of all records. There is limited data for year 2014. Therefore, the year 2014 is dropped and the three-year data from 2015 to 2017 comprised the study cohort. The data fields of BCBS claims data are shown in Table 10. Note that paid amount is not perfectly correlated with the actual cost for the requested procedure, as it may include additional services such as radiologists' fees and facility fees. Additionally, UT pays BCBS $10 per member per month to subscribe to the BVA services. According to BCBS, the BVA program saved $27,020,735 for UT during the study period. However, this statistic is found unreliable because BCBS calculates savings only when beneficiaries choose a lower-cost provider after consulting with agents, excluding cases where beneficiaries select a higher-cost provider, which would have resulted in negative savings.

A preliminary analysis yields two key insights: first, beneficiaries tend to choose higher-cost providers; second, BVA agents usually guide beneficiaries towards lower-cost providers. These findings are illustrated using call records from the Houston area requesting MRI services. These patterns hold consistently across other regions and other procedures as well. Panel 1 of Fig 3 displays requested amount versus recommended amount. The average cost of a beneficiary's initially requested providers is significant higher than that of providers recommended by agents (paired t-test, $p < 0.001$). Panel 2 focus on a single commonly requested MRI procedure, MRI of the lower limb joint without contrast (13.66% of all MRI requests). It displays the frequency with which providers from different price quartiles are requested by beneficiaries versus recommended by agents. Beneficiaries typically request providers in the third or fourth quartile of prices, that is, higher-cost providers, while agents predominantly recommend providers in the first price quartile, i.e., lower-cost providers. As expected, the BVA agents direct patients to lower-cost providers subject to location and travel considerations, which is reflected in the observation that they also recommend high-cost providers, but much less frequently (14% of the time). Note that the provider location and quality information are not available in the data.

S2 Fig 3. (1) Requested versus Recommended amount. (2) Percentage of times providers belonged to different price quartile by requested versus recommended. The price quartile is determined based on requested amount.

The claims data have three key limitations necessitating a behavioral experiment. First,

Data Element	Explanation & Value		
Employee location	Nine cities in Texas: Arlington, Austin, Dallas, EI Paso, San Antonio, Tyler, Houston, Odessa, Edinburg.		
BVA agent number	116 unique agent IDs in the data		
Row id	40,137 claims in the data		
Procedure group	20 procedure groups in total. Top 3 procedure groups: MRI (22,386, 55.77%), CT Scan (10,097, 25.16%), Office Visit (1,704, 4.25%)		
Procedure name	396 different procedure names		
Call date	The date of the call ranges from 09/09/2014 to 08/31/2018		
Service date	The date of the actual health service happens, ranging from 09/01/2015 to 08/31/2018		
Shoppable procedure flag	Binary variable which takes value 1 if the procedure is shoppable		
Requested amount	The amount of requested provider, ranging from $0[†] to $186,793		
Recommended amount	The amount of recommended amount, ranging from $3 to $186,793		
Paid amount	The actual amount paid by the employer ranging from $0 to $322,139.17		
Saving actions	Beneficiaries actual choices, which could be the requested provider, recommended provider, or a provider that is less (or more) expensive than the requested one.		
Year[††] (frequency, percentage)	2014 (1,532, 3.82%), 2015 (11,134, 27.74%), 2016 (12,952, 32.27%), 2017 (14,519, 36.17%)		
**Avg. (std.) of Requested, Recommended and Paid Amounts for Key Procedures[†††]**			
	Requested amount ($)	Recommended amount ($)	Paid amount ($)
MRI lower spine without contrast	2179 (1275)	1248 (1025)	2806 (5582)
MRI lower limb joint without contrast	1164 (617)	653 (465)	977 (895)
MRI Brain without and with Contrast	1197 (738)	641 (498)	1249 (2255)
CAT Scan Head/Brain without Contrast	646 (441)	336 (350)	895 (5929)
CAT Scan Chest without and with Contrast	1291 (743)	723 (598)	1781 (3261)

Notes: [†] When requested amount equals 0, the beneficiary does not have a preferred provider when calling agents. [††] In practice, provider price is negotiated on an annual basis. Price are typically updated at the beginning of September. The year is defined according to the following criteria: Year 2014: call date from 09/01/2014 to 08/31/2015; so on and so forth. [†††] Key procedures include top 3 most frequently requested MRI procedures and top 2 most frequently requested CT Scan procedures.

Table 10. Data Summary

the data do not include beneficiaries' actual choices of providers, as beneficiaries' selected provider IDs are not observed. Additionally, detailed patient demographics are not observed. This lack of information limits the possibility of a thorough econometric analysis. Second, all treatments - copay waivers, persuasion, and recommendations - are applied simultaneously by agents, making it challenging to isolate the individual and incremental effects of each strategy on beneficiaries' decisions. Third, the claims data are subject to self-selection bias. Only about 14% of patients utilize the BVA program, and their participation may be influenced by UT's offer of a copay waiver for those who call. The observed patterns result from two distinct but interdependent factors - participation bias and provider preferences, making it difficult to generalize the findings to a broader population. Finally, the goal is to assess the relative effectiveness of each strategy to help practitioners better manage and promote the program. To address these limitations, an online behavioral experiment is designed based on BCBS' implementation of the BVA program for UT, using a more diverse sample. This approach allows to investigate the research questions in a more generalizable context.
