# Supplementary material for: An evaluation of strategies commonly used by health advocate programs
Source: PLoS One. 2026 Jul 17;21(7):e0350645. doi: 10.1371/journal.pone.0350645 (PMC13379028; doi:10.1371/journal.pone.0350645)
Supplement: S3 File — Payment structure in the BVA program. (PDF) [file pone.0350645.s009.pdf]

S3 Appendix. Payment Structure in the BVA Program

The study focuses on non-urgent diagnostic procedures for which beneficiaries typically pay a fixed copayment amount or a proportional coinsurance amount. The insurer pays the negotiated price minus the beneficiaries' share. Depending on the health plan and the type of service, the beneficiaries may also pay a specified sum across all such services, called the deductible amount, before the insurance coverage kicks in. The beneficiaries' share of costs is referred to as their out-of-pocket cost. For example, most diagnostic tests for UT Select beneficiaries do not have a co-insurance component. Instead, beneficiaries pay a fixed co-pay (e.g., $100), which varies by service but is independent of the amount charged by the service provider-this is what the study models. Other payment structures, such as co-insurance, are possible but fall outside the scope of the study, which is based on the BCBS BVA program. Furthermore, under a co-insurance regime, where patients' out-of-pocket costs are proportional to the service provider's charges, the need for a BVA program diminishes, as patients are naturally incentivized to select lower-priced providers.
