# Supplementary material for: An evaluation of strategies commonly used by health advocate programs
Source: PLoS One. 2026 Jul 17;21(7):e0350645. doi: 10.1371/journal.pone.0350645 (PMC13379028; doi:10.1371/journal.pone.0350645)
Supplement: S4 File — Price negotiations for non-urgent and shoppable procedures & misperception of price-quality relationship. (PDF) [file pone.0350645.s010.pdf]

S4 Appendix. Price Negotiations for Non-urgent and Shoppable Procedures & Misperception of Price-Quality Relationship

The BVA program neither limits beneficiaries' choices nor directs them to inferior options. It targets frequently prescribed non-urgent and shoppable medical procedures. Industry-standard processes for performing such procedures are well-established, resulting in minimal variation in quality across providers. Providers' prices are predominantly determined by their market presence and bargaining leverage when negotiating with insurers (Hussey et al. 2013). Factors such as provider size, uniqueness of services, market concentration, and level of competition further influence this leverage (Trish and Herring 2015, Jones and Mills 2006). There is anecdotal evidence from the UT Employee Benefits Office that hospitals negotiate prices on a basket of services, for instance, bundling diagnostic procedures like MRIs with different types of common orthopedic procedures such as knee joint replacement. Insurers focus on the weighted average cost across these service packages, taking into account patient volume and service utilization. For example, one hospital may offer low prices for MRI procedures but charge high rates for other services included in the basket, while another might do the opposite to remain competitive. As a result, prices for the same procedure negotiated by the same insurer with different providers can vary significantly (Whaley 2015). Similarly, different insurers' negotiated prices for the same procedure (such as an MRI) performed at the same hospital can also vary dramatically (Ericson 2022). In contrast, free-standing diagnostic facilities offer a limited menu of services, resulting in less negotiation leverage and typically lower prices.

The perception that higher price equates to higher quality is not necessarily true for providers of shoppable medical procedures, which is the central premise of the BVA program. The program attempts to direct beneficiaries to lower-cost providers, putting pressure on providers to lower prices without compromising beneficiaries' choice. In the long term, beneficiaries benefit from a financially healthy system. They should experience a slower growth in healthcare premiums if the costs of routine high-volume procedures could be lowered. In addition to cost and quality, agents' recommendations also consider location and beneficiaries' travel constraints. For example, based on a beneficiary's input, agents may filter providers located within a certain distance threshold from the beneficiary's residence or work place and only recommend providers that belong to this selected group. The experiment also incorporates this feature.

References

1. Ericson KM. Negotiating lower hospital prices as a self-insured employer. Harvard Business Review. 2022 May 3. Available at https://hbr.org/2022/05/negotiating-lower-hospital-prices-as-a-self-insured-employer, Accessed April 14, 2026.

2. Hussey PS, Wertheimer S, Mehrotra A. The association between health care quality and cost: A systematic review. Annals of Internal Medicine. 2013;158(1):27-34.

3. Jones CL, Mills Jr TL. Negotiating a contract with a health plan. Family Practice Management. 2006;13(10):49-55.

4. Trish EE, Herring BJ. How do health insurer market concentration and bargaining power with hospitals affect health insurance premiums? Journal of Health Economics. 2015;42:104-114.
5. Whaley C. Searching for health: The effects of online price transparency. 2015;DOI:10.2139/SSRN.2684809.
