# Supplementary material for: An evaluation of strategies commonly used by health advocate programs
Source: PLoS One. 2026 Jul 17;21(7):e0350645. doi: 10.1371/journal.pone.0350645 (PMC13379028; doi:10.1371/journal.pone.0350645)
Supplement: S5 File — The survey instrument. (PDF) [file pone.0350645.s011.pdf]

S5 Appendix. The Survey Instrument

Section A: Consent Form

Please read this consent form before giving your consent.

In this form you will find information that may affect your decision as to whether or not to participate in this research study. Read the information below and ask any questions you might have before deciding whether or not to take part.

Purpose of the Study: The study aims to investigate how beneficiaries choose among providers of shoppable medical services (MRI, CT Scans, etc.). You are invited to make choices among healthcare providers under various situations.

What is your role? If you agree to participate in this study, you will be asked to participate in three consecutive components.

- Component 1 will consist of a one-page web-based tutorial on MRI procedures and demographic questions. We expect Session A to take about 2 minutes
- Component 2 is a short quiz on the healthcare insurance terms, which is composed of one to two questions. Right answers will be revealed automatically if you choose the wrong answer. We expect Session B to take about 3-4 minutes.
- Component 3 is a web-based survey. You will be asked to follow the instructions in the survey and answer questions. There are no right or wrong answers in this session and the situations presented to you are entirely hypothetical. You will see multiple healthcare provider options under various scenarios and you will be asked to make a choice. We expect Session C to take about 5 minutes.

Are there risks? There are no foreseeable risks in participating in this study. The risk associated with this study is no greater than everyday life. This study entails the minimal risk of the loss of privacy and/or confidentiality of responses. Strong precautions against this loss of privacy and confidentiality will betaken and thus the likelihood of this risk is minimal. Should an unanticipated problem arise, the researcher in charge will contact the appropriate university authority immediately.

Are there benefits? Through the study, you will gain knowledge of the Benefits Value Advisor (BVA) service, which may facilitate your decisions in the future. In addition, your response will help us better understand the impact of BVA program on patient choices.

Will there be any compensation? Upon completion of the three sessions (i.e. A, B and C), you will receive $2. We create HITs for the survey and prepay to MTurk via credit card. After you finish the survey and complete a HIT, you will get paid by MTurk. At the end of survey, you will see your secrete code. Note that only when you finish all three Sessions (i.e. A, B and C) and submit your secrete code correctly, you can get the compensation. In addition, MTurk does not allow for prorated compensation. If you enter Session B or C but have an incomplete response, you will NOT get the compensation.

Do you have to participate? No, your participation is entirely voluntary. You may decide not to participate at all or, if you start the study and then change your mind,

you may stop participating at any time by closing the browser window or the program to withdraw from the study. Withdrawal or refusing to participate will not affect you in any respect. Your responses will be destroyed and not counted in the analysis of survey data if you withdraw during the experiment. It is worth noting that once we approve your HIT and Amazon process your payment, you may NOT withdraw your participation anymore.

Privacy and confidentiality The only identifying information based on your participation in this study will be your Amazon Mechanical Turk serial number. We note that this could be linked to your public profile page, so you might consider what information you choose to share on your public profile. Your Mechanical Turk Worker ID will be used to distribute payment to you but will NOT be stored with the research data we collect from you. We note again that the serial numbers will only remain with Amazon; we will code your transcriptions with a new random number for our data collection purposes. We will NOT be accessing any personally identifying information about you that you may have put on your Amazon public profile page.

Your response to the survey is anonymous. Access to the response information will be limited to the investigators and co-investigators and is strictly limited to use in academic research, including publications in peer-reviewed journals and presentations at professional meetings. If the results of this research are published or presented at scientific meetings, only aggregated statistical information will be included. All the information will be safely stored on password protected computers that may only be accessed by authorized researchers. All data will be destroyed by secure means no later than 5 years after the completion of the study.

If it becomes necessary for the Institutional Review Board to review the study records, information that can be linked to you will be protected to the extent permitted by law. Your research records will not be released without your consent unless required by law or a court order. The data resulting from your participation may be made available to other researchers in the future for research purposes not detailed within this consent form. In these cases, the data will contain no identifying information that could associate it with you, or with your participation in any study. Any researcher who may be granted access to the data will be required to observe the protocols contained in this consent form.

Whom to contact with questions about the study? At any time, you can contact the main researchers involved in this study: Diwakar Gupta, diwakar.gupta@mccombs.utexas.edu and Jingyao Huang, jingyao.huang@mccombs.utexas.edu.The researchers will make every effort to clarify any questions you might have about the study.

Whom to contact with questions concerning your rights as a research participant? For questions about your rights or any dissatisfaction with any part of this study, you can contact,anonymously if you wish, the Institutional Review Board by phone at (512) 471-8871 or email atorsc@uts.cc.utexas.edu. If you are interested, a research participants' bill of right is presented at

https://research.utexas.edu/resources/human-subjects/for-research-participants.
Participation If you agree to participate, please click on “Agree. I consent to participate in this study.” button which indicates that (1) You have read the above information, (2) You voluntarily agree to participate, and (3) You are 25 years of age or older.

- Agree. I consent to participate in this study.
- Disagree. I do not wish to participate in this study.

Section B: Background Knowledge
What is an MRI scan? An Magnetic resonance imaging (MRI) is a diagnostic test that uses powerful magnets, radio waves, and a computer to make detailed pictures inside your body (Fig 4). Your doctor can use this test to diagnose you or to see how well you've responded to treatment. If you are interested, a more detailed introduction to MRI procedures is provided at https://www.medicalnewstoday.com/articles/146309.

S5 Fig 4. An example of MRI Scan. Computer-generated image by the authors using Claude. Note that the figure is similar but not identical to the image we used in the survey and is therefore for illustrative purposes only.

Section C: Demographic Questions Q1 - Q8 are provided in **S6 Appendix**.
Section D: Quiz
Subjects are randomly assigned to either Q9(1), or Q9(2).
Q9[1]. Suppose your doctor recommends that you get an MRI done. Based on the recommendations of your doctor and/or your family and friends, you think that provider 1 is a good choice. According to your health insurer's web site, the cost of provider 1 is $2000. Your health plan requires a $100 copay for MRI procedures. If you choose provider 1. what is your out-of-pocket cost for an MRI?
A. $0 B.$100 C. $200 D. $2000 E. Don't know
Q9[2]. Suppose your doctor recommends that you get an MRI done. Based on the recommendations of your doctor and/or your family and friends, you think that provider 1 is a good choice. According to your health insurer's web site, the cost of provider 1 is $2000. Your health plan requires a $100 co-pay for MRI procedures no matter what provider you choose. Recently, your insurer has launched a new service: Benefits Value Advisor(BVA). If you call the BVA, you will be given information about cost and quality of providers within 3 miles distance from your residence. The BVA agent may recommend a provider for you. Also, if you call BVA, you will get the $100 co-pay waived. No matter which provider you choose ultimately, you will get the waiver if you call BVA. In other words, you do not have to follow the recommendation by BVA to get the co-pay waiver. You decide to try the new service and called BVA. The consultant gives you a lot of suggestions and recommended another provider, say, provider 2. The cost of provider 2 is also $2000. After speaking to the consultant, you still want to go to provider 1, then what is your out-of-pocket cost for an MRI if you choose provider 1?
A. $0 B.$100 C. $200 D. $2000 E. Don't know

Q10[1]. (Subjects got Q10[1] only if they didn't answer Q9[1] correctly) Suppose your doctor recommends that you get an MRI done. Based on the recommendations of your doctor and/or your family and friends, you think that provider 2 is a good choice. According to your health insurer's web site, the cost of provider 2 is $2050. Your health plan requires a $100 copay for MRI procedures. If you choose provider 2, what is the cost to your insurer?

A. $50 B. $100 C. $1900 D. $1950 E.Don't know

Q10[2]. (Subjects got Q10[2] only if they didn't answer Q9[2] correctly) After speaking to the consultant, you think provider 2 may be a better choice, then what is your out-of-pocket cost if you choose provider 2?

A. $0 B.$100 C. $200 D. $2000 E. Don't know

Section E. Winograd Check (Each subject was randomly assigned to one Winograd question).

Q11. The lawyer asked the female witness a question, but he was reluctant to repeat it. Who was reluctant to repeat the question? Please answer with a single word and the first letter NOT capitalized.

Q12. The firemen arrived before the police woman because she was coming from far away. Who came from far away? Please answer with a single word and the first letter NOT capitalized.

Q13. There is a pillar between me and the stage, and I cannot see it. What cannot I see? Please answer with a single word and the first letter NOT capitalized.

Q14. I poured water from the bottle into the cup until it was empty. What was empty? Please answer with a single word and the first letter NOT capitalized.

Section F. Scenarios

Background description: Suppose you have a medical condition for which your doctor has recommended that you get an MRI done. Note that your condition is neither life-threatening nor urgent. Your insurance plan requires you to pay a $100 copay for the MRI. You are thinking about going to the Orange Clinic for your MRI, which is close to where you live (within 3 miles). Recently, your insurer has launched a new service: Benefits Value Advisor (BVA). If you call the agent, you will be given information about cost and quality of providers within 3 miles distance from your residence.

Each subject was randomly assigned to one scenario.

Scenario 1: Q15(1): You decide to try the new service and call the agent. The consultant provides you with information on four clinics below, which are all within 3 miles distance from your residence (Fig 5). Among these clinics is the Orange Clinic where you initially thought you would like to go. Your agent informs you that the four clinics have the same out-of-pocket cost to you. *Which provider would you choose?*

S5 Fig 5. Provider Information

Scenario 2: Q15(2): You decide to try the new service and call the agent. The consultant provides you with information on four clinics below. which are all within 3

miles distance from your residence (Fig 6). Among these clinics is the Orange Clinic where you initially thought you would like to go. Your agent informs you that the four clinics have the same out-of-pocket cost to you. In addition, the BVA consultant recommends the Pear Clinic for you. *Which provider would you choose?*

S5 Fig 6. Provider Information with Recommendation

Scenario 3: Q15(3): You decide to try the new service and call the agent. The consultant provides you with information on four clinics below, which are all within 3 miles distance of your residence (Fig 7). Among these clinics is the Orange Clinic where you initially thought you would like to go. After speaking to the consultant, you learn that because you tried the BVA service, your insurer has decided to offer you a $100 copay waiver. No matter which clinic you choose ultimately, you will get the $100 waived and your out-of-pocket cost will be $0. Now all the information you acquired is listed in the table below. Your agent informs you that the four clinics have the same out-of-pocket cost to you. *Which provider would you choose?*

S5 Fig 7. Provider Information with Copay Waiver

Scenario 4: Q15(5): You decide to try the new service and call the agent. The consultant provides you with information on four clinics, which are all within 3 miles from your residence (Fig 5). Among these clinics is the Orange Clinic where you initially thought you would like to go. Your agent informs you that the four clinics have the same out-of-pocket cost to you. Before you make a choice, the agent shares with you an email that the program received from a beneficiary. *"Hi. My name is Ashley, I am a graduate student. Unfortunately, I had a meniscal tear during a basketball game two weeks ago and was required to get an MRI scan. My friend suggested that I go to some clinic, which I am going to call Clinic A. Meanwhile, I happen to learn about the BVA program, which can provide information and suggestions. I decided to call the agent for some advice and the agent provided me some options and quality information on those healthcare providers. After the conversation, I decided to choose Clinic B, which is a radiology lab that is much less expensive than Clinic A. I was apprehensive at first, but Clinic B turned out to be a great experience. They were very professional. The staff was friendly and nice. I finished my MRI scan on time. I am very satisfied with this experience. —Ashley" Which provider would you choose?*

Scenario 5: Q15(4): You decide to try the new service and call the agent. The BVA consultant provides you the information on four clinics below, which are all 3 miles from your residence (Fig 8). Among these clinics is the Orange Clinic where you initially thought you would like to go. In addition, the BVA consultant recommends the Pear Clinic for you. After speaking to the consultant, you learn that because you tried the BVA service, your insurer has decided to offer you a $100 copay waiver. No matter which clinic you choose ultimately, you will get the $100 waived. Now all the

information you acquired is listed in the table below. Your agent informs you that the four clinics have the same out-of-pocket cost to you. *Which provider would you choose?*

S5 Fig 8. Provider Information with Copay Waiver & Recommendation

Scenario 6: Q15(6): You decide to try the new service and call the agent. The consultant provides you with information on four clinics below, which are all within 3 miles from your residence (Fig 6). Among these clinics is the Orange Clinic where you initially thought you would like to go. Your agent informs you that the four clinics have the same out-of-pocket cost to you. In addition, the BVA consultant recommends the Pear Clinic for you. Before you make a choice, the agent would like to share with you an email that the program received from a beneficiary.
"Hi. My name is Ashley, I am a graduate student. Unfortunately, I had a meniscal tear during a basketball game two weeks ago and was required to get an MRI scan. My friend suggested that I go to some clinic, which I am going to call Clinic A. Meanwhile, I happen to learn about the BVA program, which can provide information and suggestions. I decided to call the agent for some advice and the agent provided me some options and quality information on those healthcare providers. After the conversation, I decided to choose Clinic B, which is a radiology lab that is much less expensive than Clinic A. I was apprehensive at first, but Clinic B turned out to be a great experience. They were very professional. The staff was friendly and nice. I finished my MRI scan on time. I am very satisfied with this experience. — Ashley" Which provider would you choose?

Scenario 7: Q15(7): You decide to try the new service and call the agent. The consultant provides you with information on four clinics below, which are all within 3 miles distance from your residence (Fig 7). Among these clinics is the Orange Clinic where you initially thought you would like to go. After speaking to the consultant, you learn that because you tried the BVA service, your insurer has decided to offer you a $100 copay waiver. No matter which clinic you choose ultimately, you will get the $100 waived. Now all the information you acquired is listed in the table below. Your agent informs you that the four clinics have the same out-of-pocket cost to you. Before you make a choice, the BVA would like to share with you an email that the program received from a beneficiary. *"Hi. My name is Ashley, I am a graduate student. Unfortunately, I had a meniscal tear during a basketball game two weeks ago and was required to get an MRI scan. My friend suggested that I go to some clinic, which I am going to call Clinic A. Meanwhile, I happen to learn about the BVA program, which can provide information and suggestions. I decided to call the agent for some advice and the agent provided me some options and quality information on those healthcare providers. After the conversation, I decided to choose Clinic B, which is a radiology lab that is much less expensive than Clinic A. I was apprehensive at first, but Clinic B turned out to be a great experience. They were very professional. The staff was friendly and nice. I finished my MRI scan on time. I am very satisfied with this experience. — Ashley" Which provider would you choose?*

Scenario 8: Q15(8) You decide to try the new service and call the agent. The BVA consultant provides you the information on four clinics, which are all within 3 miles distance from your residence (Fig 8). Among these clinics is the Orange Clinic where you initially thought you would like to go. In addition, the BVA consultant recommends the Pear Clinic for you. Through your conversation with the consultant, you learn that because you tried the BVA service, your insurer has decided to offer you a $100 copay waiver. No matter which clinic you choose ultimately, you will get the $100 waived. Now all the information you acquire is listed in the table below. Your agent informs you that the four clinics have the same out-of-pocket cost to you. Before you make a choice, the agent would like to share with you an email that the program received from a beneficiary. *"Hi. My name is Ashley, I am a graduate student. Unfortunately, I had a meniscal tear during a basketball game two weeks ago and was required to get an MRI scan. My friend suggested that I go to some clinic, which I am going to call Clinic A. Meanwhile, I happen to learn about the BVA program, which can provide information and suggestions. I decided to call the agent for some advice and the agent provided me some options and quality information on those healthcare providers. After the conversation, I decided to choose Clinic B, which is a radiology lab that is much less expensive than Clinic A. I was apprehensive at first, but Clinic B turned out to be a great experience. They were very professional. The staff was friendly and nice. I finished my MRI scan on time. I am very satisfied with this experience. — Ashley" Which provider would you choose?*

Subjects were asked to provide a brief reason for their slection at the end of each scenario.

Section G. Final Question

Q16. Please indicate how strongly you agree with the following statement.

I trust my insurance company. 1-Strongly agree, 2-Agree, 3-Somewhat agree, 4 Neither agree or disagree, 5-Somewhat disagree, 6-Disagree, 7-Strongly disagree".
