# Supplementary material for: An evaluation of strategies commonly used by health advocate programs
Source: PLoS One. 2026 Jul 17;21(7):e0350645. doi: 10.1371/journal.pone.0350645 (PMC13379028; doi:10.1371/journal.pone.0350645)
Supplement: S6 File — Sample size selection & mturk experiment completion rate. (PDF) [file pone.0350645.s012.pdf]

S6 Appendix. Sample Size Selection & MTurk Experiment Completion Rate

A minimum sample size requirement of 351 was determined using the GPower 3.1 software following guidelines provided in Faul et al. (2009), which utilized a two-tailed test with 85% power to detect an odds ratio of 2, assuming no other predictors in the model and a 5% risk of Type I error. Subsequently, we considered the possibility of attrition in the subject pool, variations in the realized effect size, and the possible inclusion of additional covariates. These considerations led us to evaluate the impact of raising the target sample size to 500. Upon performing further tests, the target sample size of 500 yielded an ex-post power of 99% for two-tailed test and an odds ratio of 2.85, assuming a 5% risk of Type I error and a modest contribution (4%) of other covariates to the outcome variance. These tests were instrumental in the selection of the final sample size of 500.

Once the task was launched on MTurk platform, it automated the data collection process. Subjects were paid $2 for completing the survey which was estimated to take 10-15 minutes. The bounce rate of the study was 2.75%. The bounce rate measures the percentage of MTurk workers who were qualified for the study and previewed the assignment but decided not to participate. Among those MTurkers who agreed to participate, the completion rate in the study was 88.75%, which would be considered high for studies of this nature (the average completion rate has been reported to be less than 80% – see, e.g. Roddy and Robinson 2021) and would indicate high data quality and reliability (Eysenbach 2004).

Reference

1. Eysenbach G. Improving the quality of web surveys: The checklist for reporting results of internet E-surveys (CHERRIES). Journal of Medical Internet Research. 2004;6(3):e132.

2. Faul F, Erdfelder E, Buchner A, Lang AG. Statistical power analyses using G*Power 3.1: Tests for correlation and regression analyses. Behavior Research Methods. 2009;41(4):1149-1160.

3. Roddy J, Robinson S. An exploration of stress: Leveraging online data from crowdsourcing platforms. Frontiers in Artificial Intelligence. 2021;4:591-529.
