# Supplementary material for: An evaluation of strategies commonly used by health advocate programs
Source: PLoS One. 2026 Jul 17;21(7):e0350645. doi: 10.1371/journal.pone.0350645 (PMC13379028; doi:10.1371/journal.pone.0350645)
Supplement: S7 File — Results of Preliminary model – logit regression. (PDF) [file pone.0350645.s013.pdf]

S7 Appendix. Results of Preliminary Model - Logit Regression

**M1: Choosing the lowest-cost provider**									
	(1)	(2)	(3)	(4)	(5)	(6)	(7)	(8)	(9)
**Recommendation**	1.223*** (0.196)	1.233*** (0.196)	1.246*** (0.197)	1.284*** (0.199)	1.300*** (0.202)	1.328*** (0.204)	1.326*** (0.206)	1.333*** (0.206)	1.333*** (0.209)
**Copay Waiver**	-0.472 (0.193)	-0.047 (0.193)	-0.046 (0.193)	-0.103 (0.195)	-0.109 (0.198)	-0.098 (0.199)	-0.124 (0.201)	-0.140 (0.202)	-0.141 (0.204)
**Persuasion**	-0.302 (0.193)	-0.300 (0.193)	-0.303 (0.193)	-0.314 (0.196)	-0.323 (0.198)	-0.326 (0.199)	-0.350 (0.200)	-0.346 (0.202)	-0.321 (0.204)
Gender	No	Yes	Yes	Yes	Yes	Yes	Yes	Yes	Yes
Insurance	No	No	Yes	Yes	Yes	Yes	Yes	Yes	Yes
Income	No	No	No	Yes	Yes	Yes	Yes	Yes	Yes
Age	No	No	No	No	Yes	Yes	Yes	Yes	Yes
Race	No	No	No	No	No	Yes	Yes	Yes	Yes
Education	No	No	No	No	No	No	Yes	Yes	Yes
Employment Status	No	No	No	No	No	No	No	Yes	Yes
English Proficiency	No	No	No	No	No	No	No	No	Yes
Observations	498	498	498	493	493	491	491	491	482
Pseudo R^2	0.0653	0.0672	0.0689	0.0746	0.0874	0.0912	0.099	0.1019	0.1084
**M2: Choosing the lower-cost provider**									
	(1)	(2)	(3)	(4)	(5)	(6)	(7)	(8)	(9)
**Recommendation**	0.969*** (0.187)	0.981*** (0.188)	0.991*** (0.189)	1.022*** (0.190)	1.038*** (0.192)	1.049*** (0.194)	1.043*** (0.196)	1.046*** (0.196)	1.037*** (0.199)
**Copay Waiver**	-0.028 (0.187)	-0.028 (0.187)	-0.027 (0.187)	-0.087 (0.190)	-0.086 (0.191)	-0.077 (0.192)	-0.094 (0.194)	-0.109 (0.195)	-0.110 (0.197)
**Persuasion**	-0.235 (0.187)	-0.232 (0.187)	-0.235 (0.187)	-0.246 (0.189)	-0.248 (0.191)	-0.248 (0.193)	-0.278 (0.194)	-0.267 (0.195)	-0.239 (0.197)
Gender	No	Yes	Yes	Yes	Yes	Yes	Yes	Yes	Yes
Insurance	No	No	Yes	Yes	Yes	Yes	Yes	Yes	Yes
Income	No	No	No	Yes	Yes	Yes	Yes	Yes	Yes
Age	No	No	No	No	Yes	Yes	Yes	Yes	Yes
Race	No	No	No	No	No	Yes	Yes	Yes	Yes
Education	No	No	No	No	No	No	Yes	Yes	Yes
Employment Status	No	No	No	No	No	No	No	Yes	Yes
English Proficiency	No	No	No	No	No	No	No	No	Yes
Observations	498	498	498	493	493	491	491	491	482
Pseudo R^2	0.0425	0.0452	0.0465	0.0516	0.0623	0.0663	0.0747	0.0777	0.0829

Notes: $^{***}p < 0.01$, $^{**}p < 0.05$, $^{*}p < 0.1$.
The numbers on the first row in each cell are the coefficients of regression results, and the numbers on the second row are the standard deviations.

Table 11. Regression Results for the Preliminary Model **(Logit Regression)**.
