# Supplementary material for: An evaluation of strategies commonly used by health advocate programs
Source: PLoS One. 2026 Jul 17;21(7):e0350645. doi: 10.1371/journal.pone.0350645 (PMC13379028; doi:10.1371/journal.pone.0350645)
Supplement: S9 File — Results of preliminary and comprehensive models – probit regression. (PDF) [file pone.0350645.s015.pdf]

S9 Appendix Results of Preliminary and Comprehensive Models - Probit Regression

	(1)	(2)	(3)	(4)	(5)	(6)	(7)	(8)	(9)
**M1: Choosing the lowest-cost provider**									
**Recommendation**	0.749*** (0.118)	0.758*** (0.118)	0.766*** (0.119)	0.789*** (0.120)	0.799*** (0.121)	0.812*** (0.122)	0.812*** (0.123)	0.815*** (0.123)	0.815*** (0.125)
**Copay Waiver**	-0.032 (0.117)	-0.034 (0.118)	-0.034 (0.118)	-0.075 (0.119)	-0.071 (0.120)	-0.061 (0.120)	-0.076 (0.122)	-0.085 (0.122)	-0.086 (0.123)
**Persuasion**	-0.182 (0.118)	-0.180 (0.118)	-0.182 (0.118)	-0.190 (0.119)	-0.199 (0.120)	-0.199 (0.120)	-0.218 (0.121)	-0.217 (0.122)	-0.202 (0.123)
Gender	No	Yes	Yes	Yes	Yes	Yes	Yes	Yes	Yes
Insurance	No	No	Yes	Yes	Yes	Yes	Yes	Yes	Yes
Income	No	No	No	Yes	Yes	Yes	Yes	Yes	Yes
Age	No	No	No	No	Yes	Yes	Yes	Yes	Yes
Race	No	No	No	No	No	Yes	Yes	Yes	Yes
Education	No	No	No	No	No	No	Yes	Yes	Yes
Employment Status	No	No	No	No	No	No	No	Yes	Yes
English Proficiency	No	No	No	No	No	No	No	No	Yes
Observations	498	498	498	493	493	491	491	491	482
Pseudo R^2	0.0653	0.0674	0.0691	0.0758	0.0882	0.0918	0.1000	0.1028	0.1091
**M2: Choosing the lower-cost provider**									
	(1)	(2)	(3)	(4)	(5)	(6)	(7)	(8)	(9)
**Recommendation**	0.601*** (0.115)	0.609*** (0.116)	0.615*** (0.116)	0.634*** (0.117)	0.642*** (0.118)	0.646*** (0.119)	0.642*** (0.119)	0.643*** (0.119)	0.633*** (0.121)
**Copay Waiver**	-0.019 (0.115)	-0.020 (0.115)	-0.021 (0.115)	-0.058 (0.117)	-0.056 (0.117)	-0.049 (0.118)	-0.058 (0.119)	-0.066 (0.119)	-0.066 (0.120)
**Persuasion**	-0.143 (0.115)	-0.141 (0.115)	-0.143 (0.115)	-0.149 (0.116)	-0.151 (0.117)	-0.152 (0.118)	-0.172 (0.118)	-0.166 (0.119)	-0.145 (0.120)
Gender	No	Yes	Yes	Yes	Yes	Yes	Yes	Yes	Yes
Insurance	No	No	Yes	Yes	Yes	Yes	Yes	Yes	Yes
Income	No	No	No	Yes	Yes	Yes	Yes	Yes	Yes
Age	No	No	No	No	Yes	Yes	Yes	Yes	Yes
Race	No	No	No	No	No	Yes	Yes	Yes	Yes
Education	No	No	No	No	No	No	Yes	Yes	Yes
Employment Status	No	No	No	No	No	No	No	Yes	Yes
English Proficiency	No	No	No	No	No	No	No	No	Yes
Observations	498	498	498	493	493	491	491	491	482
Pseudo R^2	0.0424	0.0452	0.0465	0.0516	0.0624	0.0664	0.0749	0.0779	0.0826

Notes: $^{***}p < 0.01$, $^{**}p < 0.05$, $^{*}p < 0.1$.
The numbers on the first row in each cell are the coefficients of regression results, and the numbers on the second row are the standard deviations.

Table 14. Regression Results for the Preliminary Model **(Probit Regression)**.

M1: Choosing the lowest-cost provider									
	(1)	(2)	(3)	(4)	(5)	(6)	(7)	(8)	(9)
**Recommendation**	0.920*** (0.218)	0.927*** (0.218)	0.947*** (0.219)	0.957*** (0.221)	0.940*** (0.225)	0.980*** (0.229)	0.971*** (0.230)	0.953*** (0.231)	0.908*** (0.233)
**CopayWaiver**	-0.223 (0.219)	-0.225 (0.219)	-0.223 (0.220)	-0.278 (0.222)	-0.290 (0.223)	-0.255 (0.225)	-0.252 (0.225)	-0.284 (0.227)	-0.343 (0.230)
**Persuasion**	-0.188 (0.222)	-0.186 (0.222)	-0.178 (0.222)	-0.168 (0.223)	-0.186 (0.225)	-0.172 (0.230)	-0.179 (0.231)	-0.203 (0.232)	-0.261 (0.236)
**Recommendation + CopayWaiver**	0.136 (0.244)	0.137 (0.244)	0.134 (0.245)	0.169 (0.247)	0.190 (0.250)	0.147 (0.254)	0.137 (0.255)	0.161 (0.256)	0.200 (0.259)
**Recommendation + Persuasion**	-0.091 (0.244)	-0.090 (0.244)	-0.093 (0.244)	-0.101 (0.246)	-0.083 (0.249)	-0.107 (0.253)	-0.089 (0.254)	-0.065 (0.256)	0.010 (0.261)
**CopayWaiver + Persuasion**	0.105 (0.242)	0.103 (0.243)	0.093 (0.243)	0.082 (0.246)	0.092 (0.248)	0.088 (0.249)	0.063 (0.251)	0.077 (0.252)	0.126 (0.255)
**Mistrust**	-0.037 (0.373)	-0.034 (0.372)	-0.042 (0.374)	-0.010 (0.376)	0.026 (0.380)	0.000 (0.383)	-0.027 (0.385)	-0.017 (0.385)	-0.014 (0.389)
**Recommendation + Mistrust**									
1 *Do Not Mistrust (base level)*									
1 *Mistrust*	−0.785** (0.341)	−0.801** (0.342)	−0.852** (0.346)	−0.854** (0.351)	−0.834** (0.354)	−0.812** (0.358)	−0.789** (0.359)	−0.814** (0.360)	−0.872** (0.364)
**CopayWaiver + Mistrust**									
1 *Do Not Mistrust (base level)*									
1 *Mistrust*	0.294 (0.340)	0.291 (0.340)	0.288 (0.342)	0.259 (0.346)	0.225 (0.349)	0.222 (0.357)	0.232 (0.359)	0.263 (0.360)	0.266 (0.364)
**Persuasion + Mistrust**									
1 *Do Not Mistrust (base level)*									
1 *Mistrust*	0.003 (0.336)	0.006 (0.335)	-0.008 (0.338)	-0.068 (0.342)	-0.107 (0.347)	-0.108 (0.351)	-0.097 (0.349)	-0.085 (0.352)	-0.068 (0.354)
**Pass**	0.851*** (0.174)	0.835*** (0.176)	0.820*** (0.176)	0.804*** (0.178)	0.768*** (0.179)	0.742*** (0.183)	0.781*** (0.180)	0.731*** (0.183)	0.623*** (0.190)
Gender	No	Yes	Yes	Yes	Yes	Yes	Yes	Yes	Yes
Insurance	No	No	Yes	Yes	Yes	Yes	Yes	Yes	Yes
Income	No	No	No	Yes	Yes	Yes	Yes	Yes	Yes
Age	No	No	No	No	Yes	Yes	Yes	Yes	Yes
Race	No	No	No	No	No	Yes	Yes	Yes	Yes
Education	No	No	No	No	No	No	Yes	Yes	Yes
Employment Status	No	No	No	No	No	No	No	Yes	Yes
English Proficiency	No	No	No	No	No	No	No	No	Yes
Observations	498	498	498	493	493	491	491	491	482
Pseudo R^2	0.1203	0.1210	0.1238	0.1300	0.1378	0.1415	0.1454	0.1473	0.1492

Notes: $^{***}p < 0.01$, $^{**}p < 0.05$, $^{*}p < 0.1$.
The numbers on the first row in each cell are the coefficients of regression results, and the numbers on the second row are the standard deviations.

Table 15. Regression Results for the Comprehensive Model of M1 **(Probit Regression)**.

M2: Choosing the lower-cost provider									
	(1)	(2)	(3)	(4)	(5)	(6)	(7)	(8)	(9)
**Recommendation**	0.742***<br>(0.212)	0.751***<br>(0.212)	0.769***<br>(0.213)	0.781***<br>(0.215)	0.773***<br>(0.219)	0.815***<br>(0.222)	0.800***<br>(0.223)	0.776***<br>(0.224)	0.739***<br>(0.227)
**CopayWaiver**	-0.184<br>(0.210)	-0.187<br>(0.210)	-0.185<br>(0.335)	-0.230<br>(0.212)	-0.239<br>(0.213)	-0.206<br>(0.215)	-0.196<br>(0.215)	-0.217<br>(0.217)	-0.263<br>(0.220)
**Persuasion**	-0.065<br>(0.211)	-0.063<br>(0.211)	-0.057<br>(0.212)	-0.050<br>(0.212)	-0.056<br>(0.214)	-0.034<br>(0.218)	-0.040<br>(0.219)	-0.056<br>(0.220)	-0.093<br>(0.223)
**Recommendation<br>+ CopayWaiver**	0.202<br>(0.236)	0.203<br>(0.236)	0.201<br>(0.237)	0.226<br>(0.239)	0.241<br>0.241	0.189<br>(0.244)	0.189<br>(0.246)	0.210<br>(0.247)	0.239<br>(0.250)
**Recommendation<br>+ Persuasion**	-0.192<br>(0.235)	-0.192<br>(0.235)	-0.193<br>(0.236)	-0.198<br>(0.238)	-0.195<br>(0.240)	-0.229<br>(0.243)	-0.212<br>(0.244)	-0.177<br>(0.246)	-0.124<br>(0.250)
**CopayWaiver<br>+ Persuasion**	0.074<br>(0.235)	0.074<br>(0.235)	0.062<br>(0.236)	0.054<br>(0.238)	0.066<br>(0.239)	0.060<br>(0.241)	0.022<br>(0.242)	0.020<br>(0.243)	0.066<br>(0.247)
**Mistrust**	0.086<br>(0.362)	0.087<br>(0.362)	0.082<br>(0.363)	0.115<br>(0.365)	0.144<br>(0.367)	0.120<br>(0.370)	0.102<br>(0.372)	0.117<br>(0.372)	0.146<br>(0.376)
**Recommendation + Mistrust**									
1 *Do Not Mistrust (base level)*									
1 *Mistrust*	−0.726**<br>(0.333)	−0.747**<br>(0.333)	−0.798**<br>(0.337)	−0.802**<br>(0.342)	−0.782**<br>(0.344)	−0.758**<br>(0.347)	−0.745**<br>(0.349)	−0.770**<br>(0.350)	−0.847**<br>(0.354)
**CopayWaiver + Mistrust**									
1 *Do Not Mistrust (base level)*									
1 *Mistrust*	-0.005<br>(0.334)	-0.009<br>(0.334)	-0.014<br>(0.335)	-0.047<br>(0.339)	-0.079<br>(0.342)	-0.068<br>(0.349)	-0.064<br>(0.351)	-0.034<br>(0.352)	-0.043<br>(0.357)
**Persuasion + Mistrust**									
1 *Do Not Mistrust (base level)*									
1 *Mistrust*	-0.040<br>(0.329)	-0.036<br>(0.329)	-0.045<br>(0.331)	-0.101<br>(0.334)	-0.127<br>(0.339)	-0.133<br>(0.341)	-0.127<br>(0.343)	-0.117<br>(0.344)	-0.090<br>(0.347)
**Pass**	0.603***<br>(0.158)	0.577***<br>(0.160)	0.564***<br>(0.160)	0.547***<br>(0.162)	0.515***<br>(0.163)	0.534***<br>(0.164)	0.493***<br>(0.167)	0.482***<br>(0.167)	0.369***<br>(0.175)
Gender	No	Yes	Yes	Yes	Yes	Yes	Yes	Yes	Yes
Insurance	No	No	Yes	Yes	Yes	Yes	Yes	Yes	Yes
Income	No	No	No	Yes	Yes	Yes	Yes	Yes	Yes
Age	No	No	No	No	Yes	Yes	Yes	Yes	Yes
Race	No	No	No	No	No	Yes	Yes	Yes	Yes
Education	No	No	No	No	No	No	Yes	Yes	Yes
Employment Status	No	No	No	No	No	No	No	Yes	Yes
English Proficiency	No	No	No	No	No	No	No	No	Yes
Observations	498	498	498	493	493	491	491	491	482
Pseudo R^2	0.0797	0.0812	0.0838	0.0892	0.0969	0.1011	0.1061	0.1081	0.1097

Notes: $^{***}p < 0.01$, $^{**}p < 0.05$, $^{*}p < 0.1$.
The numbers on the first row in each cell are the coefficients of regression results, and the numbers on the second row are the standard deviations.

Table 16. Regression Results for the Comprehensive Model of M2 **(Probit Regression)**.
