# Supplementary material for: An evaluation of strategies commonly used by health advocate programs
Source: PLoS One. 2026 Jul 17;21(7):e0350645. doi: 10.1371/journal.pone.0350645 (PMC13379028; doi:10.1371/journal.pone.0350645)
Supplement: S10 File — Additional analysis involving winograd Check. (PDF) [file pone.0350645.s016.pdf]

S10 Appendix. Additional Analysis involving Winograd Check

The study employs a Winograd attention check to ascertain whether subjects are focused during the survey. The results are reported in Table 17. There is a strong correlation between the indicator variable on good understanding and binary variable of whether subjects pass the attention check. There is a greater proportion of subjects passing the quiz on health insurance cost-sharing if they pass the Winograd check, compared to those who failed the Winograd check.

(1) Winograd Check				Pass the quiz?	
Pass Winograd check?	Frequency	Percentage	Pass Winograd check?	Yes	No
Yes	396	79.52%	Yes	350	46
No	102	20.48%	No	53	49
			(2) Correlation: Pearson chi2(1) = 69.7026 p = 0.000		

Table 17. Summary of Winograd Check

[1]Only subjects who passed the Winograd Check			[2]Control the variable indicating whether subjects pass Winograd Check (denoted as WinoSuccess)		
	M1	M2		M1	M2
**Recommendation**	1.309*** (0.436)	1.295*** (0.433)	**Recommendation**	1.504*** (0.392)	1.210*** (0.376)
**CopayWaiver**	-0.674 (0.424)	-0.437 (0.411)	**CopayWaiver**	-0.631 (0.391)	-0.464 (0.367)
**Persuasion**	-0.448 (0.430)	-0.217 (0.418)	**Persuasion**	-0.399 (0.393)	-0.160 (0.368)
**Mistrust**	-0.440 (0.715)	-0.202 (0.689)	**Mistrust**	-0.003 (0.638)	0.227 (0.610)
**Recommendation + Mistrust**			**Recommendation + Mistrust**		
1 *Do Not Mistrust (base level)*			1 *Do Not Mistrust (base level)*		
1 *Mistrust*	−1.362** (0.679)	−1.285* (0.660)	1 *Mistrust*	−1.437*** (0.607)	−1.385** (0.582)
**Pass**	0.762* (0.417)	0.514 (0.394)	**Pass**	0.947*** (0.337)	0.589* (0.306)
Gender	Yes	Yes	Gender	yes	Yes
Insurance	Yes	Yes	Insurance	Yes	Yes
Income	Yes	Yes	Income	Yes	Yes
Age	Yes	Yes	Age	Yes	Yes
Race	Yes	Yes	Race	Yes	Yes
Education	Yes	Yes	Education	Yes	Yes
Employment Status	Yes	Yes	Employment Status	Yes	Yes
English Proficiency	Yes	Yes	English Proficiency	Yes	Yes
			**WinoGrad Success**	0.306 (0.298)	0.031 (0.279)
Observations	385	385	Observations	482	482
Pseudo R^2	0.1456	0.1273	Pseudo R^2	0.1502	0.1100

Table 18. Logit regression analysis of M1 & M2, considering Winograd check results. In the first regression, the model was run on subjects who passed the Winograd check. In the second regression, WinoSuccess was controlled. For simplicity, we did not include interactions that are insignificant. $^{***}p < 0.01$, $^{**}p < 0.05$, $^{*}p < 0.1$
