# Supplementary material for: An evaluation of strategies commonly used by health advocate programs
Source: PLoS One. 2026 Jul 17;21(7):e0350645. doi: 10.1371/journal.pone.0350645 (PMC13379028; doi:10.1371/journal.pone.0350645)
Supplement: S11 File — Oversampling results. (PDF) [file pone.0350645.s017.pdf]

S11 Appendix. Oversampling Results

To address the issue of imbalanced data, where fewer respondents mistrust their insurance company, an oversampling technique is employed to validate the results. To mitigate overfitting concerns, the minority class (respondents expressing mistrust) is progressively oversampled at twice, three times, four times, and five times its original size, with the regression model re-estimated at each increment. The key results remain qualitatively consistent throughout.

	Oversampling Multiples of the Minority Group Sample Size			
	**M1: choosing the lowest-cost provider**			
	2 Times	3 Times	4 Times	5 Times
**REC**	1.519*** (0.346)	1.537*** (0.335)	1.556*** (0.325)	1.573*** (0.317)
**COP**	-0.392 (0.356)	-0.384 (0.346)	0.374 (0.338)	-0.363 (0.331)
**PER**	-0.130 (0.354)	0.001 (0.343)	0.107 (0.334)	0.197 (0.326)
**REC + COP**	0.364 (0.380)	0.458 (0.357	0.530 (0.339)	0.586* (0.322)
**REC + PER**	0.024 (0.374)	-0.452 (0.354)	-0.091 (0.376)	-0.648 (0.319)
**COP + PER**	0.024 (0.374)	-0.097 (0.351)	-0.195 (0.333)	-0.277 (0.317)
**MISTRU**	-0.036 (0.461)	-0.034 (0.398)	-0.032 (0.363)	-0.031 (0.340)
**REC + MISTRU**				
1 *Do Not Mistrust (base level)*				
1 *Mistrust*	−1.283*** (0.432)	−1.274*** (0.376)	−1.266*** (0.345)	−1.259*** (0.325)
**COP + MISTRU**				
1 *Do Not Mistrust (base level)*				
1 *Mistrust*	0.494 (0.428)	0.499 (0.372)	0.502 (0.342)	0.505 (0.322)
**PER + MISTRU**				
1 *Do Not Mistrust (base level)*				
1 *Mistrust*	-0.027 (0.421)	-0.030 (0.367)	-0.033 (0.337)	-0.035 (0.318)
**Pass**	1.383*** (0.292)	1.359*** (0.283)	1.338*** (0.276)	1.318*** (0.270)
Observations	576	654	732	810
Pseudo R^2	0.1105	0.1037	0.0987	0.0948

Notes: $^{***}p < 0.01$, $^{**}p < 0.05$, $^{*}p < 0.1$.
The numbers on the first row in each cell are the coefficients of regression results, and the numbers on the second row are the standard deviations.

Table 19. Logit Regression Analysis of M1 with Full Controls Using Oversampling Data

	Oversampling Multiples of the Minority Group Sample Size			
	**M2: choosing the lower-cost providers**			
	2 Times	3 Times	4 Times	5 Times
**REC**	1.180*** (0.333)	1.164*** (0.321)	1.156*** (0.312)	1.151*** (0.305)
**COP**	-0.303 (0.335)	-0.305 (0.325)	-0.305 (0.318)	-0.303 (0.311)
**PER**	0.028 (0.333)	0.127 (0.323)	0.208 (0.315)	0.275 (0.308)
**REC + COP**	0.488 (0.362)	0.610 (0.341)	0.705** (0.324)	0.781** (0.309)
**REC + PER**	-0.412 (0.360)	-0.493 (0.339)	−0.562* (0.322)	-0.620 (0.307)
**COP + PER**	-0.048 (0.360)	-0.169 (0.339)	-0.266 (0.322)	-0.346 (0.307)
**MISTRU**	0.161 (0.441)	0.161 (0.380)	0.161 (0.346)	0.160 (0.323)
**REC + MISTRU**				
1 *Do Not Mistrust (base level)*				
1 *Mistrust*	−1.189*** (0.414)	−1.182*** (0.360)	−1.177*** (0.330)	−1.174*** (0.311)
**COP + MISTRU**				
1 *Do Not Mistrust (base level)*				
1 *Mistrust*	-0.004 (0.414)	0.003 (0.360)	0.007 (0.330)	0.011 (0.310)
**PER + MISTRU**				
1 *Do Not Mistrust (base level)*				
1 *Mistrust*	-0.071 (0.407)	-0.070 (0.355)	-0.069 (0.326)	-0.068 (0.307)
**Pass**	1.000*** (0.259)	1.005*** (0.253)	1.010*** (0.247)	1.014*** (0.241)
Observations	576	654	732	810
Pseudo R^2	0.0766	0.0744	0.0730	0.0719

Notes: $^{***}p < 0.01$, $^{**}p < 0.05$, $^{*}p < 0.1$.
The numbers on the first row in each cell are the coefficients of regression results, and the numbers on the second row are the standard deviations.

Table 20. Logit Regression Analysis of M2 with Full Controls Using Oversampling Data
