# Supplementary material for: An evaluation of strategies commonly used by health advocate programs
Source: PLoS One. 2026 Jul 17;21(7):e0350645. doi: 10.1371/journal.pone.0350645 (PMC13379028; doi:10.1371/journal.pone.0350645)
Supplement: S12 File — External validity. (PDF) [file pone.0350645.s018.pdf]

S12 Appendix. External Validity

Behavioral studies using lab experiments need to address the concern that beneficiaries faced with similar choices in real settings may not make the same selections. The external validity of the experiment reported in this paper can be argued as follows. The analysis shows that the patterns observed are statistically unlikely to result from random choices. Additionally, the analysis compares subjects' provider selection with the beneficiaries' behavior in the claims data. Although the actual providers selected by beneficiaries are not observed, it is known whether they choose a lower-cost provider compared to their requested provider. The BVA program aims to reduce medical expenses, so any choice of a cost-saving option is considered a success. For MRI procedures, 53.0% of beneficiaries select a provider who charges less than their requested provider. Since real beneficiaries in the claims data may receive recommendation, copay waiver and persuasion simultaneously, the study first fits a multinomial logit model on the experiment data, with the four providers as the dependent variable and all regression variables the same as in Eq (2) in the manuscript. Then a counterfactual analysis is conducted by setting all three treatments to 1. In the experiment, Orange Clinic is the requested provider, so either Apple Clinic or Pear Clinic is a lower-cost option than Orange Clinic. The proportion of subjects who select a lower-cost provider is 53.2%. The close match between these results provides some external validity of the findings, although the subjects in the experiment are not incentivized in the exact same way as the beneficiaries who call the BVA program.
