# Supplementary material for: An evaluation of strategies commonly used by health advocate programs
Source: PLoS One. 2026 Jul 17;21(7):e0350645. doi: 10.1371/journal.pone.0350645 (PMC13379028; doi:10.1371/journal.pone.0350645)
Supplement: S13 File — Qualitative analysis of the underlying reasons for subjects’ choices. (PDF) [file pone.0350645.s019.pdf]

S13 Appendix. Qualitative Analysis of the Underlying Reasons for Subjects' Choices The survey also asked subjects to report their reasons for selecting a particular provider after making their final decision. Their responses highlighted the multitude of motivations that influence beneficiaries choice of lowest-cost and lower-cost providers.

First, the responses reinforced the reasons why insurers like BCBS would use the three strategies used by the BVA agents. For example, in scenarios with a single strategy, over 50% of subjects who chose the lowest-cost providers cited that strategy as the key influencing factor. Beyond these direct strategic influences, we identified several other key observations. To illustrate, we present top reasons for choosing Pear Clinic and Orange Clinic (two mostly frequently selected providers) in Fig 9 excluding those directly related to recommendations, copay waivers, and persuasion.

- Subjects care about the value delivered by providers, and are willing to select lower-cost options among those with similar quality. Among the 191 subjects who selected Pear Clinic (lowest-cost provider) across eight scenarios, 59% mentioned it as the best-value provider.
- A significant proportion of subjects exhibit altruism by selecting options that reduce costs for health insurance companies. For instance, 44% chose Pear Clinic to save costs for their insurer.
- Many subjects tend to stick with their initial choice. Among the 215 subjects who selected Orange Clinic, the second-highest priced one and the requested provider, 63% cited that they preferred to stick to their initial choice.
- A substantial number of subjects believe in a positive correlation between provider prices and quality, leading them to choose expensive options. For example, 5% of subjects who chose Orange Clinic believed it had higher quality. Meanwhile, among the 69 subjects selecting Peach Clinic, the highest-cost provider, 86% believed it had the best quality.

S13 Fig 9. Top reasons for choosing Pear & Orange Clinic. Note that subjects could indicate multiple reasons for their selection, so the percentages beside the bars do not add up to 100%. Each percentage indicates the proportion of subjects who mentioned a specific reason for their choice.

These observations offer additional insights for the improvement of the BVA program. First, insurers may work to dispel the misconception that higher prices equate to higher quality, particularly for routine medical procedures. This message could be reinforced through interactions between agents and beneficiaries. Second, insurers may better align incentives by emphasizing the long-term mutual benefits for both insurers and beneficiaries. Specifically, selecting lower-cost options helps maintain the health system's financial health, leading to future premium savings for beneficiaries. BVA agents could incorporate these key points in their discussions, and insurance companies

could promote programs to educate beneficiaries. Law and Yip (2002) highlights the importance of community education and raising awareness about the function of insurance and the consequences of unnecessary health service use, which supports our findings. Note that these findings remain subject to the behavioral limitations discussed in the paper and should be interpreted as supportive rather than definitive evidence.

Reference

Law CK, Yip PSF. Acute care service utilisation and the possible impacts of a user-fee policy in Hong Kong. Hong Kong Medical Association. 2002. Available at https://hub.hku.hk/handle/10722/45497, Accessed April 14, 2026.
