# Supplementary material for: An evaluation of strategies commonly used by health advocate programs
Source: PLoS One. 2026 Jul 17;21(7):e0350645. doi: 10.1371/journal.pone.0350645 (PMC13379028; doi:10.1371/journal.pone.0350645)
Supplement: S14 File — Healthcare payment innovations. (PDF) [file pone.0350645.s020.pdf]

S14 Appendix. Healthcare Payment Innovations

Insurers in recent years have implemented several provider payment innovations to address the price variation issues. Some insurers induce providers to reduce price and improve quality by offering direct incentives. Examples include various pay-for-performance schemes in which insurers pay either a higher amount per episode or a share of the gains if either the multiple visits (re-admissions) are avoided or the cost per episode is reduced without compromising quality (Gupta et al. 2021). This approach may work well when insurers have sufficient market power to enforce implementation, such as the Centers for Medicare & Medicaid Services (CMS). Unlike CMS, commercial insurers and self-insured employers may lack the leverage to directly incentivize providers to lower prices. Therefore, they have attempted alternate approaches of which the Benefits-Value-Advisor (BVA) program is an example. In this approach, insurers steer beneficiaries toward higher-value providers, thereby putting pressure on providers to reduce prices and improve quality. Examples include BVA program, Reference Pricing (RP) and Rewards Program. In the RP scheme, the insurer sets the maximum amount, called reference price, that can be reimbursed for a procedure. If a patient selects a provider that charges more than the reference price, then the patient is responsible for the portion in excess of the reference price in addition to the copayment or the coinsurance portion of the reference price, as applicable. The Rewards Program pays a monetary reward to beneficiaries who choose a low-cost provider (Whaley et al. 2019). Unlike the RP Scheme and the Rewards Program, beneficiaries' choices do not directly influence their out-of-pocket costs under the BVA program.

References

1. Gupta D, Mehrotra M, Tang X. Gainsharing contracts for CMS' Episode-based payment models. Production and Operations Management. 2021;30(5):1290-1312.

2. Whaley CM, Vu L, Sood N, Chernew ME, Metcalfe L, Mehrotra A. Paying patients to switch: Impact of a rewards program on choice of providers, prices, and utilization. Health Affairs. 2019;38(3):440-447.
