# Supplementary material for: An evaluation of strategies commonly used by health advocate programs
Source: PLoS One. 2026 Jul 17;21(7):e0350645. doi: 10.1371/journal.pone.0350645 (PMC13379028; doi:10.1371/journal.pone.0350645)
Supplement: S15 File — Use of mechanical turk. (PDF) [file pone.0350645.s021.pdf]

S15 Appendix. Use of Mechanical Turk

This paper outlines a prospective academic study examining the effects of different treatments before individuals select a service provider. Due to ethical concerns, Institutional Review Boards typically prohibit such experiments on real patients, leading researchers to conduct similar studies in behavioral labs with university students and staff. However, many college students are under 25 years old, rely primarily on their family's insurance, and have limited exposure to healthcare systems. Additionally, achieving within-sample heterogeneity is challenging within a student population, as they often share similar demographic and socioeconomic backgrounds. The study instead used Amazon's Mechanical Turk (MTurk) to recruit subjects for several reasons. (1) MTurk has been widely used in health services, psychology, political science, and marketing studies, often yielding more representative samples than university populations (Yeh et al. 2015, Bardos et al. 2015, Bardos et al. 2016). (2) Data from MTurk are at least as reliable as those from traditional methods (Buhrmester et al. 2011, Crump et al. 2013, Kees et al. 2017). (3) MTurk has been effectively used in healthcare context, such as patient preferences (Liu et al. 2018, Hyams et al. 2021), hospital admission decisions (Kim et al. 2020), and social preferences in reimbursement negotiations (Wettstein and Boes 2021). (4) A review of 35 peer-reviewed studies confirmed MTurk as an efficient, reliable, and cost-effective tool for health and medical research, with responses comparable to conventional methods (Mortensen and Hughes 2018).

References

1. Yeh VM, Schnur JB, Margolies L, Montgomery GH. Dense breast tissue notification: Impact on women's perceived risk, anxiety, and intentions for future breast cancer screening. Journal of the American College of Radiology. 2015;12(3):261-266.

2. Bardos J, Hercz D, Friedenthal J, Missmer SA, Williams Z. A national survey on public perceptions of miscarriage. Obstetrics and Gynecology. 2015;125(6):1313.

3. Bardos J, Friedenthal J, Spiegelman J, Williams Z. Cloud based surveys to assess patient perceptions of health care: 1000 respondents in 3 days for US $300. JMIR Research Protocols. 2016;5(3):e166.

4. Buhrmester M, Kwang T, Gosling SD. Amazon's Mechanical Turk: A new source of inexpensive, yet high-quality data? Perspectives on Psychological Science. 2011;6(1):3-5.

5. Crump MJ, McDonnell JV, Gureckis TM. Evaluating Amazon's Mechanical Turk as a tool for experimental behavioral research. PLOS One. 2013;8(3):e57410.

6. Kees J, Berry C, Burton S, Sheehan K. An analysis of data quality: Professional panels, student subject pools, and Amazon's Mechanical Turk. Journal of Advertising. 2017;46(1):141-155.

7. Liu N, Finkelstein SR, Kruk ME, Rosenthal D. When waiting to see a doctor is less irritating: Understanding patient preferences and choice behavior in appointment scheduling. Management Science. 2018;64(5):1975-1996.

8. Hyams T, Golden B, Sammarco J, et al. Evaluating preferences for colorectal cancer screening in individuals under age 50 using the Analytic Hierarchy Process. BMC Health Services Research. 2021;21:754. DOI:10.1186/s12913-021-06705-9.

9. Kim SH, Tong J, Peden C. Admission control biases in hospital unit capacity management: How occupancy information hurdles and decision noise impact utilization. Management Science. 2020;66(11):5151-5170.

10. Wettstein DJ, Boes S. Assessing social preferences in reimbursement negotiations for new pharmaceuticals in Oncology: An experimental design to analyse willingness to pay and willingness to accept. BMC Health Services Research. 2021;21:234. DOI:10.1186/s12913-021-06231-8.

11. Mortensen K, Hughes TL. Comparing Amazon's Mechanical Turk platform to conventional data collection methods in the health and medical research literature. Journal of General Internal Medicine. 2018;33(4):533-538.
